# Cardiac cycle modulates alpha and beta suppression during motor imagery

**DOI:** 10.1093/cercor/bhae442

**Published:** 2024-11-22

**Authors:** Giuseppe Lai, David Landi, Carmen Vidaurre, Joydeep Bhattacharya, Maria Herrojo Ruiz

**Affiliations:** Goldsmiths, University of London, New Cross London SE14 6NW, UK; QuestIT, Via L. Cialfi 23, 53100 Siena; Basque Center on Cognition Brain and Language, Paseo Mikeletegi 69, 2°20009 Donostia San Sebastián, Gipuzkoa, Spain; Ikerbasque, Basque Foundation for Science, Plaza Euskadi, 548009 Bilbao, Spain; Berlin Institute for the Foundations of Learning and Data (BIFOLD), Straße des 17, Juni 13510623, Berlin, Germany; Goldsmiths, University of London, New Cross London SE14 6NW, UK; Goldsmiths, University of London, New Cross London SE14 6NW, UK

**Keywords:** baroreceptor hypothesis, heart-to-brain interaction, interoception, systole, diastole

## Abstract

Previous interoception research has demonstrated that sensory processing is reduced during cardiac systole, an effect associated with diminished cortical excitability, possibly due to heightened baroreceptor activity. This study aims to determine how phases of the cardiac cycle—systole and diastole—modulate neural sensorimotor activity during motor imagery (MI) and motor execution (ME). We hypothesised that MI performance, indexed by enhanced suppression of contralateral sensorimotor alpha (8–13 Hz) and beta (14–30 Hz) activity, would be modulated by the cardiac phases, with improved performance during diastole due to enhanced sensory processing of movement cues. Additionally, we investigated whether movement cues during systole or diastole enhance muscle activity. To test these hypotheses, 29 participants were instructed to perform or imagine thumb abductions, while we recorded their electroencephalography, electrocardiogram, and electromyogram (EMG) activity. We show that imaginary movements instructed during diastole lead to more pronounced suppression of alpha and beta activity in contralateral sensorimotor cortices, with no significant cardiac timing effects observed during ME as confirmed by circular statistics. Additionally, diastole was associated with significantly increased EMG on the side of actual and, to a lesser degree, imagined movements. Our study identifies optimal cardiac phases for MI performance, suggesting potential pathways to enhance MI-based assistive technologies.

## Introduction

Interoception refers to the ability of the central nervous system (CNS) to sense, interpret, integrate, and regulate information emerging from visceral organs such as the heart, gastrointestinal tract, and respiratory system (Craig 2009; Barrett and Simmons 2015; Critchley and Garfinkel 2018; Park et al. 2020). This interplay modulates a wide range of functions, including perception, cognition, emotional processing, and the sense of self, highlighting its fundamental role across various physiological and psychological domains (Critchley and Harrison 2013; Babo-Rebelo et al. 2016; Azzalini et al. 2019; Engelen et al. 2023).

The relationship between the heart and the CNS, commonly known as heart–brain interaction (HBI), is dynamic and bidirectional (Vaitl and Schandry 1995; Azzalini et al. 2019; Engelen et al. 2023). Through both descending and ascending neural pathways, this interaction modulates visceral functions and conveys organ-related information to higher-level brain areas, affecting higher-order cognitive and emotional processes (Azzalini et al. 2019; Engelen et al. 2023). Research has shown that the cardiac cycle, a central element of HBI, modulates perception, cognition, affective processing, and action, with broad implications for both basic science and clinical applications (Schulz et al. 2009; Gray et al. 2012; Garfinkel et al. 2014; Barrett and Simons 2015; Critchley and Garfinkel 2017; Khalsa et al. 2018; Khoury et al. 2018; Honda and Nakao 2022; Candia et al. 2022; Al et al. 2023; Catrambone and Valenza 2023a; Catrambone and Valenza 2023b; Candia et al. 2024). Although this modulation is considered to be partially mediated by the multisynaptic ascending pathway arising from aortic baroreceptors (Edwards et al. 2002), recent findings in the rat olfactory bulb suggest that heartbeat-induced pulsations in cerebral blood vessels can directly affect central neuronal activity through the activation of mechanosensitive channels (Jammal Salameh et al. 2024). This identifies an additional, fast mechanism of HBI that could modulate perception (Jammal Salameh et al. 2024).

Despite these advances, a notable gap remains in translational research, particularly regarding the application of these insights in clinical settings, such as neurological conditions and assistive technologies for neuromuscular disorders. Motor imagery (MI)—mentally rehearsing and focusing on the kinesthetic sensations of a movement (Savaki and Raos 2019)—is a key paradigm traditionally used in brain–computer interface (BCI) studies (Sannelli et al. 2019) and a commonly used rehabilitative exercise for poststroke survivors (Tong et al. 2017; Khan et al. 2020). It thus provides a highly relevant framework to assess HBI in these settings, with direct implications for optimizing BCI performance. Previous work showed that resting heart rate variability (HRV) explained 26% of the variance in BCI performance during a standard P300 oddball task (Kaufmann et al. 2012). More recently, an analysis of the directional time-varying interaction between electroencephalography (EEG) and HRV was implemented offline to decode MI movements (Catrambone et al. 2021). The analysis revealed that decoding accuracy was higher when signals were coupled in the direction from the brain to the heart (descending modulation). However, the detailed interplay between the cardiac cycle and sensorimotor cortices during MI, and its impact on performance remains insufficiently understood. In particular, identifying any advantage of the timing properties along the cardiac cycle on the modulation of neural activity during MI—likely representing ascending influences—could have significant implications in BCI settings, as this could be used to optimise the delivery of cues or prompts along the cardiac cycle, aiming to enhance MI outcomes and, therefore, communication.

This study aims to address this knowledge gap by investigating how the phases of the cardiac cycle—systole and diastole—affect neural sensorimotor activity during MI. We test two prevailing hypotheses based on the baroreceptor model: one suggesting the facilitation of motor actions during systole and another suggesting potential detrimental effects on sensory processing during this cardiac phase.

At each cardiac cycle, blood fills the atria during ventricular diastole, when the heart is most relaxed and is ejected into the circulatory system during ventricular systole, when the ventricles are most contracted. At the peak of systole, blood pressure reaches its highest point, triggering stretch-responsive baroreceptors located in the carotid sinus and aortic arch. These receptors fire in response to the strength and timing of cardiac contraction (Craig 2009; Critchley and Harrison 2013; Duschek et al. 2013). The baroreceptor signals are relayed to the nucleus of the tractus solitarius (NTS) in the brainstem via the vagus and glossopharyngeal nerves, regulating blood pressure and heart rate through the baroreflex (Critchley and Harrison 2013; Engelen et al. 2023). It then progresses to subcortical structures like the thalamus, amygdala, and hypothalamus, before reaching central cortical areas including the insular cortex, cingulate cortex, and primary and secondary somatosensory cortices. The effects of this ascending process can be assessed by recording neural activity with EEG alongside cardiac activity via electrocardiogram (ECG), allowing for separate analysis of neural and behavioral responses to systole and diastole (Park and Tallon-Baudry 2014; Pramme et al. 2014; Pramme et al. 2016; Bury et al. 2019; Grund et al. 2022).

How could the cardiac cycle modulate neural sensorimotor activity during MI? Two prevailing hypotheses offer contrasting effects during the most active phase of the cardiac cycle, systole, depending on whether sensory or motor processing is involved. The baroreceptor hypothesis, originally posited by Lacey (1967) and also referred to as the pulse inhibition hypothesis (Engelen et al. 2023), suggests that baroreceptor activation during systole leads to generalised cortical inhibition and attenuated sensory processing (Rau et al. 1993; Duschek et al. 2013). Direct experimental evidence from human and nonhuman animal studies supports this view, showing that neck-cuff baroreceptor stimulation can significantly reduce cortical excitability, leading to decreased muscle tone, dampened pain sensitivity, and reduced startle reflexes (Maixner 1991; Droste et al. 1994; Dworkin et al. 1994; Nyklíček et al. 2005; Duschek et al. 2013). Further evidence demonstrated that sustained stimulation of carotid sinus baroreceptors induces immobility and sleep in animals, as well as slow wave activity (Koch 1932; Bonvallet et al. 1954; referenced in Dworkin et al. 1994 and Engelen et al. 2023). This corpus of research substantiates the hypothesis that cortical activity is transiently inhibited during systole, resulting in pulsed inhibition.

Research in the somatosensory domain aligns with these findings, as systolic tactile electrical stimulation reduces stimulus perception (Motyka et al. 2019; Grund et al. 2022). Furthermore, tactile stimuli presented during systole slow down responses and require longer intervals to sample sensory information during self-initiated touches (Edwards et al. 2007; Galvez-Pol et al. 2022). Pain processing also fluctuates with the cardiac cycle, with nociceptive responses dampened during systole (Edwards et al. 2001; Edwards et al. 2002). In the auditory domain, aversive auditory stimuli at systole elicit weaker startle responses, while both visual and auditory stimuli presented during systole result in slower reaction times (Edwards et al. 2007; Schulz et al. 2009). However, results in the visual domain are mixed. Fixations predominantly occur during the diastolic phase, whereas eye saccades—less effective for detailed sensory gathering—occur more frequently during systole (Galvez-Pol et al. 2020). Moreover, some research reports enhanced visual discrimination, selection efficiency, and search capabilities during systole (Pramme et al. 2014; Pramme et al. 2016), while others find no significant effects on visual perception Park et al. (2014).

In the motor domain, a complementary hypothesis supported by additional experiments suggests a facilitatory effect of systole on motor functions. For instance, Rae et al. (2018) found that response inhibition in a stop signal task was more efficient during systole, evidenced by shorter reaction times. Detection and adaptation to performance errors in trained experts is also enhanced during systole (Bury et al. 2019). Furthermore, studies linking systole to increased self-paced oculomotor activities such as microsaccades (Ohl et al. 2016) and eye saccades (Galvez-Pol et al. 2020), along with findings that participants are more likely to initiate the presentation of visual stimuli during systole (Kunzendorf et al. 2019), suggest a tendency to initiate actions that enhance environmental sampling during this cardiac phase. These studies are particularly relevant within the active sensing framework, suggesting that individuals are more likely to initiate actions during the systolic phase to exploit heightened perceptual sensitivity in the subsequent diastolic phase, thereby gathering sensory information more efficiently (Galvez-Pol et al. 2020; Chen et al. 2021; Galvez-Pol et al. 2022).

Furthermore, recent research demonstrated that involuntary motor-evoked potentials, triggered by transcranial magnetic stimulation (TMS), were larger during systole, suggesting motor facilitation when baroreceptor activity is increased (Al et al. 2023). In a separate motor pinch task, the same study also observed more pronounced desynchronization in the alpha and beta frequency bands (8–25 Hz) when the movement onset coincided with systole rather than diastole. Earlier TMS studies did not observe similar findings (Filippi et al. 2000; Otsuru et al. 2020; Bianchini et al. 2021), possibly due to variations in sample sizes or the fixed latency of TMS application across the cardiac cycle (as argued by Al et al. 2023). Inconsistencies are further highlighted in shooting research, where nonelite rifle shooters predominantly fire during systole (Konttinen et al. 2003), while elite shooters fire more frequently during diastole, avoiding systolic pulsation (Helin et al. 1987). Additionally, studies on cognitive-emotional control have shown that cardiac cycle effects on response execution are limited to congruent trials, with faster responses and enhanced frontal theta-band (4–7 Hz) activity during diastole (Adelhöfer et al. 2020). To address these mixed findings in both the motor and sensory domains, current proposals emphasize the importance of considering the specific requirements and demands of each task, particularly how relevant the stimuli are for environmental interaction (Pramme et al. 2016; Motyka et al. 2019; Adelhöfer et al. 2020).

Given the potential implications for EEG-based BCIs, understanding the role of cardiac interoception in MI is crucial, and this includes determining its alignment with either the baroreceptor hypothesis or the proposal of motor facilitation during systole. MI in laboratory settings typically involves the processing of external cues (system prompts) and their internal rehearsal, a process that could be influenced by the cardiac cycle during cue processing and the onset of imagined movements. A systematic assessment of the phases of the cardiac cycle that enhances neural modulation in response to MI-related cues could inform the development of new BCI applications. These applications could train individuals by delivering system prompts during a specific phase of the cardiac cycle.

Both real movement execution (motor execution, ME) and its imagination activate the primary somatosensory cortex (S1) and primary motor cortex (M1) (Lotze et al. 1999; Grèzes and Decety 2001; Hétu et al. 2013; Hardwick et al. 2018), with substantial evidence of overlapping activation in M1. Since interoceptive information is integrated at central cortical levels, affecting both somatosensory and motor processes (Azzalini et al. 2019; Al et al. 2023), our primary hypothesis is that neural responses during MI—indexed by contralateral alpha and beta modulation in M1 and S1—are modulated by the phases of the cardiac cycle. More specifically, based on the evidence that somatosensory processing is attenuated during systole, we predict that MI initiated following movement cues during diastole—when sensory processing is less inhibited—would result in enhanced performance, as reflected by more pronounced contralateral alpha and beta suppression, in line with the baroreceptor hypothesis.

Increased baroreceptor firing typically inhibits muscle sympathetic nerve activity (MSNA) to stabilize blood pressure (Wallin et al. 1975; Macefield 1998). Furthermore, salient somatosensory stimulation amplifies this inhibition, enhanced by afferent baroreceptor discharge (Donadio et al. 2005). Evidence indicates that MSNA increases during upper limb muscle contractions relative to rest (Katayama and Saito 2019). Since higher MSNA has been associated with increased EMG activation during exercise (Seals and Enoka 1989), this evidence collectively suggests that increased MNSA during contractions could be suppressed by systole-timed cues. Moreover, this suppression could lead to more pronounced muscle activity, as measured by EMG, during diastole than systole.

Conversely, Al et al. (2023) observed increased EMG activation during systole in a motor pinch task, aligned with their neural findings of enhanced alpha and beta suppression. Accordingly, whether EMG activation of movements is enhanced or suppressed during systole relative to diastole remains undetermined. Muscle spindles modulate their discharge in response to arterial pulsations (Birznieks et al. 2012); however, it is unclear if this modulation would influence EMG activation when movements are cued or initiated during systole or diastole. Here, we aimed to determine if muscle activity, measured via EMG during cues for ME, is attenuated during systole or enhanced during this phase, relative to diastole. We anticipate that any residual EMG activity during MI will show similar, albeit less pronounced, modulation as observed during ME.

To test our hypotheses, we conducted an experiment where 29 participants performed a ME task, followed by an MI task involving the same movements, while we recorded their synchronized EEG, ECG, and EMG activity. These tasks required participants to perform or imagine left and right thumb abductions, guided by the direction of an arrow displayed on the screen. Our results reveal that imaginary movements instructed during diastole lead to more pronounced suppression of alpha and beta activity in contralateral sensorimotor cortices, with no cardiac timing effects observed during ME. Additionally, we observed increased EMG activity on the side of both imagined (MI) and actual (ME) movements during diastole. These findings identify optimal cardiac phases for MI performance, which could inform innovative enhancements in MI-based assistive technologies.

## Materials and methods

### Participants

The study was conducted at Goldsmiths, University of London, and received ethical approval from the local ethics committee at the Department of Psychology. All participants provided written informed consent. The sample comprised 29 right-handed healthy volunteers (16 females), aged between 18 and 40 years (mean age = 20.20 years, standard error of the mean, SEM = 0.66). Participants received cash incentives or course credits for taking part in the study. Eligibility criteria included right-handedness, normal or corrected-to-normal vision, and no known history of neurological conditions. We aimed for a sample of 30 participants but excluded one post-experiment upon their disclosure of a neurological disorder, which disqualified them from the participation criteria. This sample size was informed by published effect sizes for cardiac influences on motor performance (Azevedo et al. 2017; Bury et al. 2019), which suggested Cohen’s *d* in the range 0.55–0.7 (probability of superiority of 0.69, equivalent to Cohen’s *d* ~ 0.7 in Bury et al. 2019). This implies that 24–28 participants would be sufficient to detect within-subject behavioral effects with 0.8 power.

### Experimental design

Participants were seated in a dimly lit room, facing a computer screen which displayed instructions and experimental stimuli. The experimental paradigm consisted of two tasks, preceded by an eyes-open resting-state recording lasting 5 min and an auditory oddball task (not analyzed here). The experimental paradigm was programmed using PsychoPy (Peirce et al. 2019), a Python-based open-source software toolbox designed for the presentation of visual and auditory stimuli (Python v. 3.7.11; PsychoPy v. 2021 January 4; Ubuntu v. 20.04.5 LTS—Focal Fossa).

### ME and MI

The ME and MI tasks were modeled on the protocol established by Sannelli et al. (2019). In the ME task, participants responded to the direction of an arrow displayed on the computer screen after a fixation cross by executing an abduction of either the left or right thumb. This abduction required participants to gently lift the designated thumb for approximately 1 s, maintaining it at maximum extension until the arrow reverted to the fixation cross, which occurred 4 s after the arrow. Importantly, participants were asked to focus their attention on the kinesthetic aspects of the movement such as muscle contraction and movement velocity. The kinesthetic sensation was defined as any sensation experienced during the execution of the movement that the participant felt salient. In the MI task, participants were instructed to mentally recreate the kinesthetic experience of the movement without physical execution or visualization, ensuring consistency in sensations and similar timing throughout the task.

**Fig. 1:**
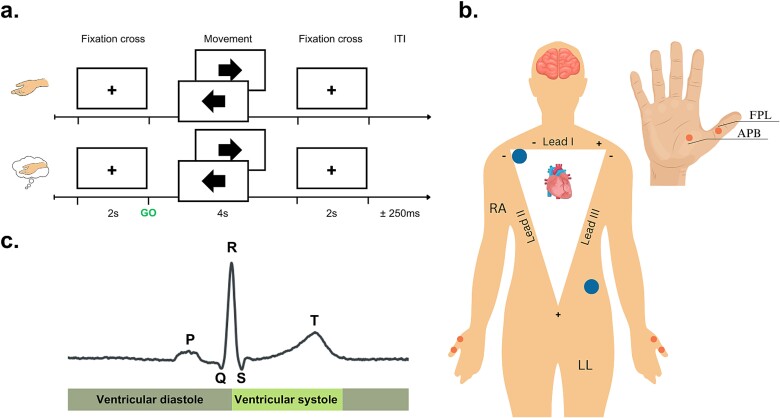
Illustration of the experimental paradigm. a) The experiment consisted of the ME task, where participants performed either a left or right thumb abduction movement based on the direction of the arrow. This movement lasted for approximately 1 s after the presentation of the left or right arrows. For the subsequent 3 s, participants were required to maintain the thumb in the lifted position and then relax once the fixation cross reappeared on the screen. b) The diagram shows the locations of the ECG electrodes (indicated by blue dots) based on the lead-II system configuration and the placements of the EMG electrodes (shown as orange dots). One electrode was placed on the flexor pollicis longus (FPL) and the other one on the abductor pollicis brevis (APB). c) A schematic representation demonstrating the key events in the ECG trace, along with definitions for the phases of the cardiac cycle, specifically the ventricular diastole and ventricular systole.

As shown in Fig.1a, each trial lasted approximately 8 s. The ME task consisted of a single block of 50 trials (25 left and 25 right), with an automatic 15-s pause after the first 25 trials. For the MI task, participants completed two blocks of 100 trials each, with an automatic 15-s pause every 25 trials and a break between the two blocks. The number of left and right trials was evenly distributed (100 trials for each condition) and their sequence was randomized.

### E‌EG, ECG, and EMG recordings

EEG data were collected using the BioSemi ActiveTwo (BioSemi Inc.) with a 64-channel layout based on the 10–20 electrode-placement system and were recorded at a sampling rate of 1024 Hz. Two external electrodes were positioned on the left and right mastoids as an initial reference upon importing the data. ECG data were recorded using the lead II configuration: the negative electrode was positioned under the right collar bone, and the positive electrode was placed above the left hip bone (Fig.1b). Additionally, EMG data from both the left and right thumbs were recorded using four bipolar electrodes, positioned on the respective abductor pollicis brevis muscle of each thumb (Fig.1b). Due to low signal-to-noise ratio in the EMG signal of three participants, their data were excluded from the main analysis. All signals were recorded using a high-pass filter at 0 Hz and a low-pass filter at 208 Hz.

### E‌EG data preprocessing

Preprocessing, epoching, and artifact rejection were carried out using the open-source Python library: MNE-Python (Gramfort et al. 2013) (Python v. 3.9.7, MNE-Python v. 1.3.0, Ubuntu v. 20.04.5 LTS—Focal Fossa). The EEG data were rereferenced to common-average reference and then band-pass filtered (zero-phase, FIR design) between 1 Hz and 40 Hz. For both ME and MI trials, data were time-locked to the onset of the arrow (or cue) on the screen and epoched between −1 and 4 s relative to the cue onset. The data were first visually inspected for rejecting large artifacts, and bad channels were interpolated using spherical splines (Perrin et al. 1989). Finally, independent component analysis, ICA (fastICA, Hyvärinen and Oja 2000) was used to remove artifact components related to eye blinks, eye saccades, and cardiac artifacts. For the identification of cardiac artifacts, we utilized the *ica.find_bads_ecg* function from MNE-Python, which is designed to identify components likely associated with ECG artifacts by correlating ICA components with the ECG signal. This function employs either cross-sectional phase statistics or Pearson correlation methods. However, this function failed to return any IC components that indicated the cardiac field artifact in any of our subjects. Accordingly, to ensure the removal of the cardiac artifact from EEG signals we used a regression-based method (https://github.com/Giuseppe-1993/HBI-motor_imagery; adapted from Esra Al’s GitHub: https://github.com/Esra-Al/Regress_ECG). A related approach has also been recently proposed (Arnau et al. 2023). A figure showing the effectiveness of this method is shown in the Supplementary Material (see Fig. S1).

### ECG data preprocessing

To remove various types of artifacts in the ECG data, such as power-line noise, muscle artifacts, electrode contact noise and low-frequency baseline drifts, filters were applied to the continuous ECG recordings. Specifically, a band-pass filter (0.2–40 Hz; zero-phase, FIR design) was employed to eliminate slow drifts and reduce the impact of muscle artifacts. To detect key cardiac events in the ECG data, we utilized NeuroKit2 (v. 0.2.4), an open-source Python library (Makowski et al. 2021). This enabled the identification of the P-wave, R-peak, and T-wave within the ECG recordings. After Azzalini et al. (2019), we defined the systolic phase as the ventricular systole, which extends from the R-peak to the T-wave offset, and the diastolic phase was defined as the ventricular diastole, spanning the interval from the T-wave offset to the next R-peak (Fig.1c).

### EMG data preprocessing

Following the recommendations set by Konrad (2005), EMG data were filtered at 10 Hz. To ensure a robust estimation of standard quantitative properties of the signal’s amplitude, such as mean, peak, minimum, and maximum, we applied a full wave rectification of the signal, which involved converting all negative values to positive ones. Additionally, we applied a smoothing function (root mean square, RMS) to retain only the mean power of the signal. This approach provided a reliable measure of muscle activation, both ipsilateral and contralateral to the cue, over time during the tasks of overt thumb abduction (ME task) and covert thumb abduction (MI task).

### ECG and EMG data analysis

Initially, we conducted a sanity check analysis of the ECG signal, examining if the average ECG profile varied between epochs associated with left-cue and right-cue, separately for ME and MI tasks. Additionally, we contrasted the inter-beat intervals (IBIs) of participants across the ME and MI tasks. In the analysis of EMG data, we aimed to determine whether the cardiac cycle modulates the difference between ipsilateral and contralateral activations separately for each motor task. Firstly, we tested the general prediction that there would be more pronounced ipsilateral activation compared to contralateral activation during ME (see review in Guillot et al. 2010). Differences in activation during MI have shown inconsistent results across studies. Some report an absence of EMG activity during MI, while others demonstrate activation of the same muscles as those used during overt muscle execution, albeit to a lesser degree (Lebon et al. 2008; Guillot et al. 2010). This phenomenon was validated using microneurographic recordings from spindle afferents innervating extensor muscles (Gandevia et al. 1997). The inconsistencies in detecting EMG activity during MI have been partly attributed to differences in the sensitivity of surface EMG sensors and the instructions of the experimental task (Guillot and Collet 2010). Crucially, protocols designed to minimize EMG during MI—while still observing robust alpha and beta suppression—have been proposed for BCI settings (Nikulin et al. 2008). These protocols mimic potential clinical applications for patients who may lack EMG activity.

As detailed in the Introduction, we aimed to determine whether muscle activity during ME—more pronounced on the ipsilateral side—would be attenuated or enhanced when the cue-related stimuli were presented during systole, relative to diastole.

Given the variable findings of EMG activity during MI—ranging from its absence to cases where it mirrors overt muscle execution to a lesser extent—we hypothesized that any detectable ipsilateral EMG activity, if more pronounced than contralateral, would also be attenuated during the presentation of cue-related stimuli in systole as compared to diastole.

### Source reconstruction of EEG signals

During MI tasks, inter-subject variability arises partly from the different strategies individuals employ when imagining movements and is further complicated by volume conduction effects in EEG, which can mask the expected laterality effects in the alpha and beta power spectral density (PSD) (Schaworonkow and Nikulin 2022; Tobón-Henao et al. 2022). While inter-subject variability may be useful to inform the development of subject-specific BCI systems, high variability can affect the results of group-level statistical analysis. In recent years, source reconstruction has emerged as a powerful technique to address inter-subject variability by estimating the cortical sources of the EEG signals, reducing the impact of volume conduction and enhancing the detection of group-level results (Tobón-Henao et al. 2022).

In our study, neural activity at the source level was reconstructed using the Linearly Constrained Minimum Variance (LCMV) beamforming technique (Van Veen et al. 1997; Sekihara and Nagarajan 2008; van Vliet et al. 2018). LCMV beamforming requires two main ingredients: the forward solution (involving head geometry and conductivity properties) and the covariance matrices (for data and noise). For the forward solution, we used a three-layer head geometry and conductivity model for a standard brain provided by MNE-Python, which employs the boundary element model method. The data-covariance and the noise-covariance matrices were estimated for each participant, utilizing a specific time window of interest (500–2000 ms postcue) for the former and a precue time window (−1000 to 0 ms) for the latter. The window of interest was selected based on the instructions provided to participants, who were directed to initiate the movement within the first second following the presentation of the cue. (see Fig. 3 for the EMG activity). This procedure resulted in 61,452 dipoles bilaterally. In an exploratory analysis, we expanded this analysis to the full window length of 4 s.

We chose to parcellate the brain using the Desikan–Killiany cortical atlas (Desikan et al. 2006), which is integrated into MNE-Python and comprises 68 brain regions bilaterally (34 per hemisphere). The motor areas in this atlas include the precentral gyrus (primary motor cortex, M1), postcentral gyrus (primary S1), and paracentral gyrus. Based on previous research on MI (Hétu et al. 2013 ; Hardwick et al. 2018), our regions of interest (ROI) from the DK atlas were the precentral gyrus (M1) and the postcentral gyrus (S1). To reduce dimensionality, the dipole-related time series were mapped to these ROI using the principal component analysis (PCA)-flip method of MNE-python, resulting in a single signal for each region and hemisphere.

The paracentral gyrus was excluded because it mainly represents lower limb movements as a combined extension of the M1 and S1 regions. The supplementary motor area (SMA) is anatomically located on the medial aspect of the superior frontal gyrus (SFG), and therefore there could be some overlap between the labeled SFG in the DKT atlas and the region where the SMA is functionally located. However, we did not include the SFG in our analysis because the SMA is not distinctly isolated in this atlas. The Destrieux Atlas (Destrieux et al. 2010) includes the SMA but is more suited for MRI/fMRI studies or EEG/MEG studies that utilize individual standard MRI images, due to its detailed labeling with 78 labels per hemisphere. This fine-grained labeling demands higher spatial resolution for accurate source reconstruction.

Although our primary EEG analysis was conducted in the source space, we supplemented it with sensor-level analysis, to assess if laterality effects were present, and whether any lateralized modulation was enhanced as a function of the cardiac cycle phases (See Supplementary Materials).

### Time–frequency decomposition

For the time-frequency analysis of EEG signals during MI and ME tasks, we employed a wavelet transform with Morlet wavelet in MNE-Python (Tallon-Baudry et al. 1997; Cohen 2019). This analysis decomposed the EEG epochs into their constituent frequency components ranging from 8 and 30 Hz, specifically alpha (8–13 Hz, also known as the mu rhythm within the MI-based BCI community; Sannelli et al. 2019) and beta (14–30 Hz) bands. The wavelet decomposition was performed on the entire duration of the epochs, spanning from −1 s to 4 s relative to the cue, using a setting of 5 cycles and a resolution of one bin per frequency (1 Hz). The resulting time-frequency PSD was baseline-corrected to the precue interval using the z-score method in MNE-Python, between −0.5 and 0 s precue, as the baseline interval. Therefore, our time–frequency analyses will provide changes in normalized PSD in units of standard deviation (SD).

### Statistical analysis

To test our hypotheses, we conducted several statistical analyses including within-subject nonparametric permutation tests and circular statistics. All statistical analyses were performed using custom scripts in Matlab (v. 2022b), Python (v. 3.9.7), and R (v. 4.2.2).

Timewise permutation tests were carried out on ECG and EMG data, to assess within-subject differences as described in “*EEG and EMG Data Analysis*”. In the source-reconstructed data, we assessed differences between ipsilateral and contralateral traces in M1 and S1 by conducting timewise permutation tests (*n* = 500 permutations). These tests contrasted the normalized PSD (power spectral density) of alpha and beta bands across both hemispheres for each sample point within the interval of interest (see below). Supplementing this analysis, on the sensor-level EEG data, we conducted cluster-based permutation tests (*n* = 500 permutations) to compare the normalized PSD of alpha and beta between left-cued and right-cued data to assess lateralization effects. We also assessed whether any lateralization effect was more pronounced as a function of systole or diastole.

Both source-domain and supplementary sensor-domain analyses were carried out first on the entire dataset including all trials regardless of cardiac information and then separately on systole and diastole trials. The primary analysis focused on data within the 500–2000 ms time window, with averaging in the alpha (8–13 Hz) and beta (14–30 Hz) frequency ranges to yield one frequency bin per band. Additionally, as an exploratory step, the analysis was extended to cover the full epoch length, from 500 to 4000 ms postcue. For all statistical analyses, we set an alpha level of 0.05 (Marozzi 2004). For two-sided permutation tests, we considered statistical values that fell in the left (2.5%) and right (97.5%) percentile of the permutation distribution (two-tailed, *P* < 0.025). In cases of multiple comparisons, we controlled the false discovery rate (FDR) at *q* = 0.05 using an adaptive linear step-up procedure (Benjamini et al. 2006), and reported the significant results using the adapted threshold *p*-value (*P*_FDR_). Nonsignificant effects after FDR control are presented simply by the p-value (*P*). Nonparametric effect sizes were estimated using the probability of superiority for dependent samples (Δ_dep_) (Grissom and Kim 2012; Ruscio and Mullen 2012).

The primary source-level EEG analysis was complemented with a control analysis to account for the varying lengths of the systole and diastole phases. Due to the longer duration of the diastole phase, more trials occurred during diastole than during systole. To address the potential effect of this imbalance on alpha and beta PSD changes, we randomly selected an equal number of trials from both the systole and diastole phases across the entire dataset. This process of random selection (with replacement) was repeated ten times—control runs. For each run, we conducted the same timewise permutation tests, applied the adaptive FDR control procedure, and estimated nonparametric effect sizes.

Next, complementing the EEG, ECG, and IBI analyses, we examined the alignment of cue latency in MI trials that exhibited more pronounced contralateral suppression in alpha and beta frequencies, relative to ipsilateral changes, using a predefined suppression threshold (see below). In both instances, we examined the specific angular positions of the cues within the cardiac cycle, analyzing continuous angles from 0 to 2π radians, instead of categorizing them into binary phases like diastole or systole. These analyses employed the Rayleigh test, which is suitable for angular data with periodic oscillations, such as cardiac activity. This test determines whether the distribution of data around a circle is nonuniform, indicating concentration at specific angles.

For the temporal alignment analysis, we converted the latency difference between the cue and the preceding R-peak into angles (θ) in radians. This conversion was done after normalizing the latency difference with the IBIs from the last four R-peaks, making the IBI estimation more robust and less sensitive to algorithmic artifacts. To do so, we used the following expression:


(1) \begin{equation*} {\theta}_i=2\pi\ \left(\frac{C_i-{R}_i}{IB{I}_i}\ \right)\kern21.75em \end{equation*}

where.

${C}_i=$ The onset of the cue.

${R}_i=$ The onset of the R-peak before the cue of interest.

${\mathrm{IBI}}_i=$ The average cardiac cycle obtained from the previous four cycles.

$i=$ The *i*^th^ trial.

Following source-domain analysis, we aimed to identify specific moments along the cardiac cycle in which contralateral suppression of the sensorimotor cortex was enhanced relative to the ipsilateral side. If successful, this would be useful in guiding follow-up BCI work, as systems prompts (e.g. left-cue) can be presented in synchrony with the optimal phases that yield maximized lateralization. To examine the continuous relationship between the timing of the cardiac cycle and the occurrence of pronounced contralateral alpha and beta suppression, we computed a trial-wise suppression index by subtracting the normalized alpha and beta PSD values of the contralateral sensorimotor side from those of the ipsilateral side for each trial (trial-wise ipsilateral minus contralateral PSD). These differences were then averaged over a significant postcue interval, identified from the primary contralateral versus ipsilateral statistical contrasts conducted independently of cardiac effects (i.e. using all trials to avoid bias in circular statistics). At the trial-by-trial level, a positive suppression index signifies trials with more pronounced contralateral suppression relative to ipsilateral suppression (the contralateral trace is more negative than the ipsilateral trace). Conversely, a negative value indicates trials with greater suppression in the ipsilateral sensorimotor cortices. After extracting a trial-wise suppression index for each participant, we analyzed the angles (θ) of trials exceeding the 50th, 75th, and 90th percentile thresholds for contralateral suppression (positive suppression index), after converting them into radians. Thresholds were also estimated in each participant. The Rayleigh test was then applied to these angular data for alpha and beta frequency bands, as well as for the M1 and S1 cortices, separately. Initial tests were conducted at the individual level to derive *p-*values and resultant vector values for single-subject effects. This was followed by a group-level analysis, using the individual mean resultant vectors, to identify any directional bias in the circular distribution of suppression-related cues on the group level. Statistical effects are provided with uncorrected *p-values* as this analysis served to explore potential time windows of clustering relevant to guide future real-time BCI studies.

Last, to determine whether the directionality effects (circular statistics) of the suppression index analysis along the cardiac cycle could be explained by randomly distributed values along the unit circle, we created a null distribution in each subject, by randomly generating angles from 0 to 2π for each trial and subject, representing random cue latencies of above-threshold power suppression values. Single-subject Rayleigh tests were followed by group-level Rayleigh tests. This analysis helped determine whether the circular distribution of randomly generated cue latencies could explain significant group-level effects, as we hypothesized for our experimental PSD suppression data.

## Results

### Assessing differences between ECG and EMG waveforms

**Fig. 2:**
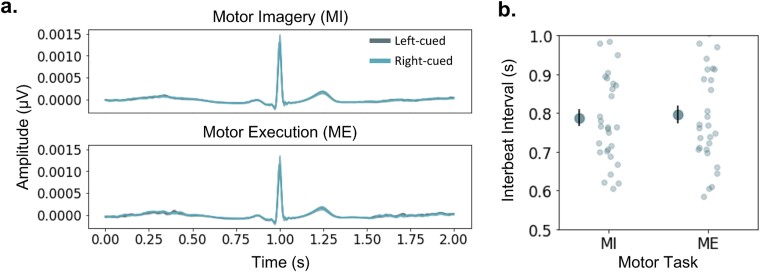
Differences between ECG waveforms and IBIs. a) The left panel shows the overlap between the ECG waveforms of left-cued (dark blue) and right-cued (light blue) in the MI (top) and ME (bottom) tasks. b) The right panel compares the IBIs between MI and ME. The larger darker dots are the mean, with the standard error of the mean represented by the vertical black bar. Lighter smaller dots are the individual data points for each participant. For both panels, nonsignificant differences were observed between the ECG waveforms across conditions (a) and participants’ IBIs (b).

In the ECG data, within-subject nonparametric permutation tests found no significant differences in the waveforms between the left-cued and right-cued trials for both MI and ME tasks (*P* > 0.05 after FDR control; see Fig. 2a). Additionally, there were no significant differences between participants’ IBIs in both motor tasks (*P* > 0.05 after FDR control; Fig. 2b).

**Fig. 3:**
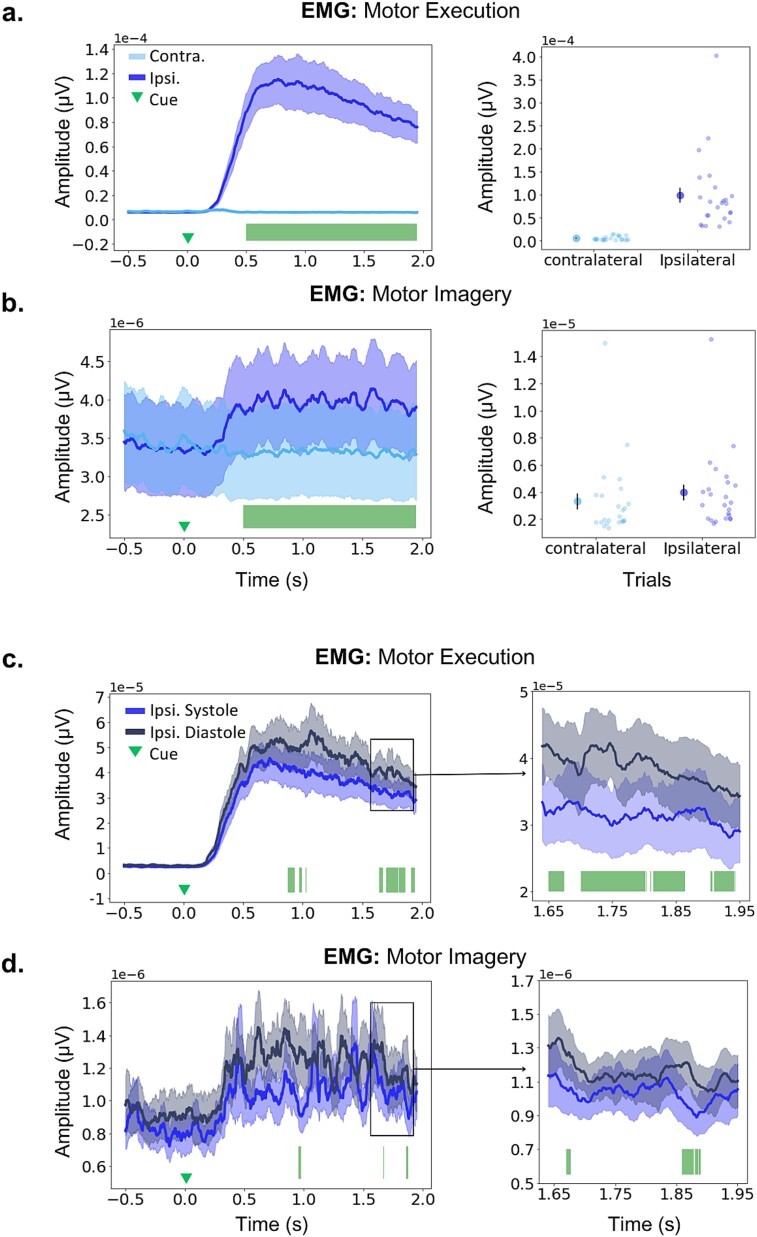
Differences between EMG waveforms. a, b) Left. Time course of ipsilateral and contralateral EMG waveforms during ME (a) and MI (b) tasks. Nonparametric permutation tests revealed a significant increase in ipsilateral EMG muscle activity, relative to contralateral, for the ME (a) and MI (b) tasks. *P* values for significant effects after FDR control were in the range *P*_FDR_ *=* 0.0001–0.0108 for ME and *P*_FDR_ *=* 0.0001 for MI. Significant effects are denoted by the green bars at the bottom. This test was conducted in the main window of interest, up until 2 s after the cue. Right. Average EMG amplitude for each participant (lighter dots) and the group mean (darker dot) obtained from contralateral and ipsilateral thumbs (the standard error of the mean, SEM, is shown with the vertical black bar). c, d) Same as a) but comparing ipsilateral EMG traces when cues instructing movement direction occur in the diastolic (darker blue) or systolic (lighter blue) phase of the cardiac cycle. Significant effects are denoted by the green bars at the bottom, with *p-values* in the range *P*_FDR_ *=* 0.0001–0.0134 for ME, *P*_FDR_ *=* 0.0001–0.002, for MI. Right: The right-side panels represent zoomed-in inserts of the EMG activity between 1.65 and 1.95 s. for all waveforms, the SEM above and below the group average is shown in lighter color areas. All statistical analyses were conducted in the subsample where EMG was available (*n* = 26), however, for illustration purposes, two participants are not included in this graphic as they had very large amplitude EMG values. Therefore, Fig. 3 represents data in *n* = 24 participants. The panels including all 26 participants can be found in Fig. S2 and S3. We did not exclude these two participants with larger EMG amplitude modulations from statistical analyses because the changes were of the same latency as in the other participants, and larger values may have been simply due to larger SNR on the day of the recording.

Subsequently, we examined the EMG differences between the left and right thumb abduction, comparing muscle activity obtained from the hand ipsilateral and contralateral to the cue (left/right arrows). This analysis was conducted separately for the MI and ME tasks (Fig. 3). We observed a significant difference in the ipsilateral minus contralateral muscle activation during ME (*P*_FDR_ = 0.0001–0.0108, range of *p-values* for time points associated with significant differences after FDR control*, Δ*_dep_ = 0.8846) and, additionally, MI (*P*_FDR_ = 0.0001*, Δ*_dep_ = 1). The significant effects extended for the entire window (500–2000 ms), as shown in Fig. 3a and b, respectively. We confirmed in a post hoc analysis that EMG activity during ME was significantly greater than during MI (*P*_FDR_ = 0.0001*, Δ*_dep_ = 1).

Next, we assessed the difference in EMG ipsilateral activation between thumb movements that occurred during systole and diastole. We found a significant difference in ME (*P*_FDR_ *=* 0.0001–0.0134, *Δ*_dep_ = 0.92; see), but also in MI (*P*_FDR_ *=* 0.0001–0.002, *Δ*_dep_ = 0.81), due to larger EMG muscle activity in diastole. The latency of the effects showed partial overlap for both tasks, occurring at approximately 0.87–1.02 s and 1.64–1.94 s during ME (Fig. 3c), and at approximately 0.95–0.97 s, around 1.67 s, and 1.85–1.88 s during MI (Fig. 3d). However, the significant effects during MI trials lasted only a few milliseconds.

### Source–domain analysis

When participants overtly executed thumb abduction, our analysis did not detect a significant lateralization effect in the postcue suppression of normalized PSD within the alpha and beta frequency ranges for both the M1 and S1 cortical regions. This absence of significant effects manifested when using all trials, as well as in the datasets of trials cued during systole and diastole (*P* > 0.05 after FDR control in all cases). No significant effects were observed in the exploratory analysis considering the full epoch length (0.5–4.0 s) either.

**Fig. 4:**
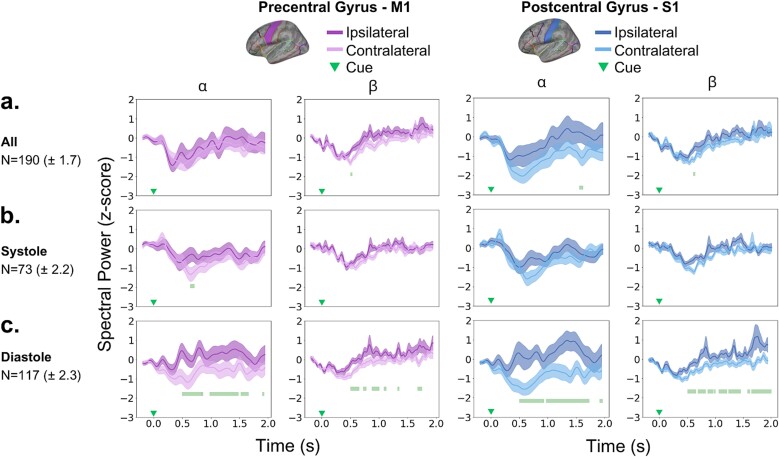
Differences between ipsilateral and contralateral power spectral density in M1 and S1. a–c) Normalized PSD in alpha and beta bands, separately in the precentral gyrus (M1, purple traces) and postcentral gyrus (S1, blue traces). Panel (a) shows the time points associated with significant differences between ipsilateral (darker color) and contralateral (lighter color) traces for all trials (*n* = 190 ± 1.7), denoted by the green bars at the bottom. Continuous lines represent the group-average, while shaded areas represent the SEM. For all trials, a significantly larger contralateral suppression was found for alpha in S1 (top left panel, *P*_FDR_ *=* 0.0001–0.0006), and for beta in M1 (top-left panel, *P*_FDR_ *=* 0.0001–0.0006) and S1 (top right panel, *P*_FDR_ *=* 0.0002–0.001). b, c) Same as a) but for systole-cued trials and diastole-cued trials, respectively. Contralateral suppression was sustained in diastole-cued trials for alpha (bottom left, M1: *P*_FDR_ *=* 0.0001–0.0544; bottom-right, S1: *P*_FDR_ *=* 0.0001–0.0592) and beta (bottom left, M1: *P*_FDR_ *=* 0.0001–0.0578; bottom-right, S1: *P*_FDR_ *=* 0.0001–0.0576) bands. In systole-cued trials, a short-lived effect was observed only in the alpha-band PSD of M1 (middle-left panel, *P*_FDR_ *= 0*.0002–0.0004).

During MI, we observed a significant lateralization of alpha and beta suppression in the dataset including all trials (Fig. 4a). This was due to more pronounced contralateral suppression in both the alpha (S1: *P*_FDR_ = 0.0001–0.0006*, Δ*_dep_ = 0.79; no effect in M1: *P* > 0.05 after FDR control) and beta bands (M1: *P*_FDR_ = 0.0001–0.0006*, Δ*_dep_ = 0.79; S1: *P*_FDR_ = 0.0002–0.001*, Δ*_dep_ = 0.76). In both regions, these significant effects were short-lived, extending for approximately 60 ms in alpha (S1: 1531–1589 ms) and 20 ms in beta (S1: 582–605 ms; M1: 500–523 ms), and therefore did not cover a full cycle at the central frequency in those bands (100 ms for 10 Hz, 50 ms for 20 Hz).

Upon separately analyzing laterality effects for trials cued during systole and diastole, in the systole-cued trial subset (Fig. 4b), significant differences were also confined to a brief time window (640–700 ms), exclusively in the alpha band and M1 region (*P*_FDR_ = 0.0002–0.0004*, Δ_dep_* = 0.79). No additional significant effects were observed for systole-cued trials (alpha, S1: *P* > 0.05 after FDR control; beta, S1 and M1: *P* > 0.05 after FDR control). Conversely, pronounced contralateral suppression effects were observed in diastole-cued trials (Fig. 4c), marked by significant differences in the alpha (M1: *P*_FDR_ = 0.0001–0.0544*, Δ*_dep_ = 0.83; S1: *P*_FDR_ = 0.0001–0.0592*, Δ*_dep_ = 0.79) and beta bands (M1: *P*_FDR_ = 0.0001–0.0578*, Δ*_dep_ = 0.86; S1: *P*_FDR_ = 0.0001–0.0576, *Δ*_dep_ = 0.83). These effects persisted throughout the entire time window, starting at 500 ms and continuing until approximately 1800–1900 ms after the onset of the S1 (alpha: 500–1931 ms; beta: 500–1953 ms) and M1 (alpha: 500–1918 ms; beta: 500–1742 ms).

Exploring potential lateralization effects over the entire epoch (0.5–4.0 s) yielded similar findings, as shown in Fig. S4. In the total dataset, significant but brief contralateral suppression occurred in both alpha (S1: P_FDR_ = 0.0004–0.0012, Δ_dep_ = 0.72) and beta (M1: P_FDR_ = 0.0001–0.0012, Δ_dep_ = 0.79; S1: P_FDR_ = 0.0004–0.0012, Δ_dep_ = 0.83) bands, with effects lasting approximately 100 and 10 ms at different times. Notably, in diastole-cued trials, significant and long-lasting contralateral suppression was observed in alpha (M1: P_FDR_ = 0.0044–0.0094, Δ_dep_ = 0.76; S1: P_FDR_ = 0.0001–0.01, Δ_dep_ = 0.9) and beta (M1: P_FDR_ = 0.0001–0.0094, Δ_dep_ = 0.86; S1: P_FDR_ = 0.0001–0.01, Δ_dep_ = 0.93) frequencies. No significant differences were obtained in systole-cued trials.

**Fig. 5:**
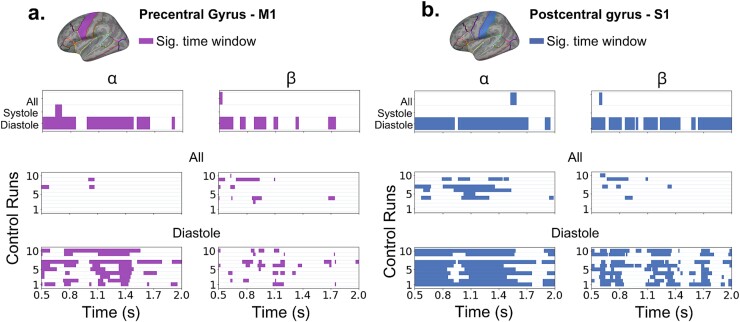
Control analysis of laterality effects by cardiac phase. The figure shows the results of the control analysis (10 runs) in the M1 (a) and S1 (b) cortical regions. The colored horizontal bars denote time windows of significant differences between contralateral and ipsilateral suppression after FDR control, and M1 (purple) and S1 (blue) regions. Top panels summarize the statistical effects of the main analysis, including all available trials of each type: Irrespective of cardiac cycle phases (“all”), systole-cued trials, diastole-cued trials. The middle panels illustrate statistical effects in the 10 control analyses conducted using a reduced number of trials, matched to the number of systole-cued trials for each participant. This revealed a few laterality effects for M1 (alpha: 2/10 runs; beta: 6/10) and S1 (alpha: 5/10; beta: 4/10), albeit in time points not overlapping with the windows showing effects in the main analysis (top panel). See range of *p*-values and effect sizes in Tables S1–S4. The bottom panels show the time intervals associated with significant laterality effects, obtained using the reduced number of diastole-cued trials. Significant effects were widespread and consistent across runs, in line with the main analysis: Significant effects observed for M1 (alpha: 9/10 runs; beta: 8/10) and S1 (alpha: 9/10; beta: 9/10).

After balancing the number of trials for diastole-cued and the full dataset based on trials in the systole-cued dataset across 10 control runs, the main effects remained consistent, as illustrated in Fig. 5. This confirmed that the observed significant and sustained contralateral suppression in alpha and beta PSD within sensorimotor cortices during diastole-cued trials was not due to a higher trial count compared to systole-cued trials. Refer to Tables S1–S4 for the corresponding statistics. Indeed, even when considering all trials, regardless of the cardiac phase, the effect was less sustained, as reported above (Fig. 4).

**Fig. 6:**
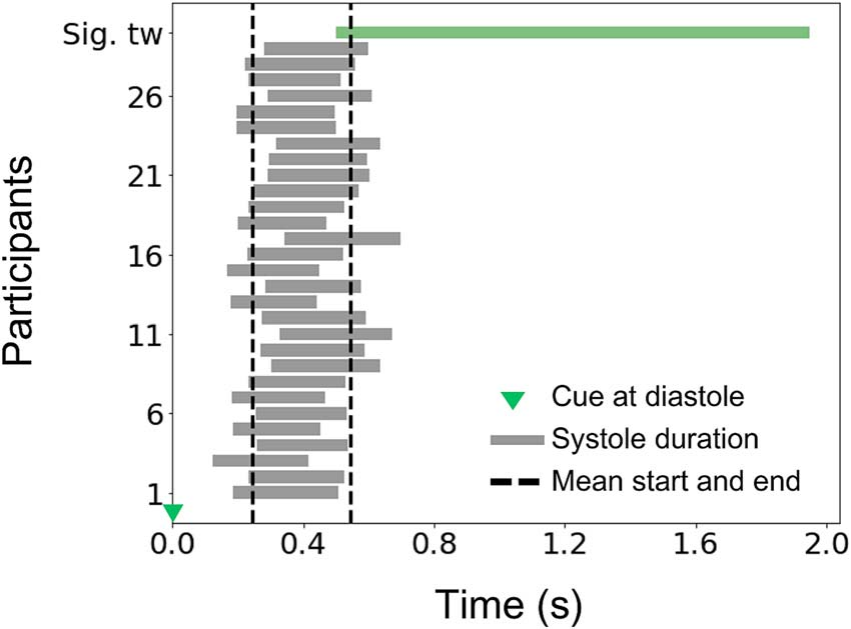
Latency of significant lateralization effect of alpha and beta suppression in diastole-cued trials. The onset of significant lateralization effects in S1 and M1 around 0.5 s (extending during 500–1900 ms, denoted by the green bar on top) did not consistently overlap with the duration of the subsequent systolic phase (mean duration: 243–546 ms) for each participant. On average, the overlap was for 46 ms only. Time zero coincides with the presentation of the cue during the diastolic phase.

Last, although a robust and widespread significant lateralization effect of alpha and beta suppression was observed when the cue was aligned with the diastolic phase, we conducted a visual inspection to determine if the onset of significant effects coincided with the systole phase. This post hoc analysis was motivated by recent work indicating heightened motor cortex excitability when TMS is administered during systole, as well as during a motor pinch task (Al et al. 2023). As shown in Fig. 6, when cues were presented during diastole, the onset of significant suppression effects did not consistently align with the systole phase across participants. Furthermore, the significant effects lasted from 0.5 to 1.9 s, indicating that the enhanced contralateral suppression observed in diastole-cued trials extends across various phases of the cardiac cycle, transitioning from diastole to systole.

### Sensor-domain analysis

As a supplementary analysis (see Supplementary Materials), we conducted a cluster-based permutation test to compare the suppression of baseline-corrected alpha and beta PSD between left- and right-cued conditions to identify any lateralization effects during the execution of real and imagery movements. No significant lateralization was detected during ME. However, in the MI task, the analysis of all trials revealed significant differences in alpha and beta suppression when contrasting between left-cued and right-cued trials, due to a contralateral suppression for left-cued trials yet bilateral suppression for right-cued trials (*P*_FWER_ = 0.002; Fig. S5). A similar significant effect was obtained for trials cued during diastole (*P*_FWER_ = 0.014), due to more contralateral suppression to left-cued trials (bilateral for right-cued trials). No significant effects were observed in trials cued during systole (*P*_FWER_ = 0.2036–0.9122). For the analysis including all trials, the latency of the significant effect spanned from 500 to 2000 ms, and from 500 to 1000 ms for trials cued during diastole (see Fig. S5 in the Supplementary Materials).

A post hoc exploratory analysis assessing alpha and beta suppression specifically in selected contralateral and ipsilateral sensorimotor EEG electrodes revealed more pronounced contralateral than ipsilateral suppression when cues were aligned with the diastole phase during MI (beta-band effect: *P* < 0.01, uncorrected exploratory analysis). No laterality effects were observed during systole. See Figs. S6 and S7. In a further exploratory analysis during ME, there were no laterality effects detected when considering either systole or diastole trials (Figs. S8 and S9).

### Position of the cue within the cardiac cycle and contralateral suppression.

We employed circular statistics to evaluate whether the latency of cues in trials with particularly pronounced source-level contralateral alpha and/or beta suppression clustered at a specific angle on the unit circle, representing the cardiac cycle. This process involved initially calculating the contralateral suppression index (PSD ipsilateral—contralateral) for each trial and participant. Positive suppression index values indicate more contralateral suppression, while negative values denote greater ipsilateral suppression.

**Fig. 7:**
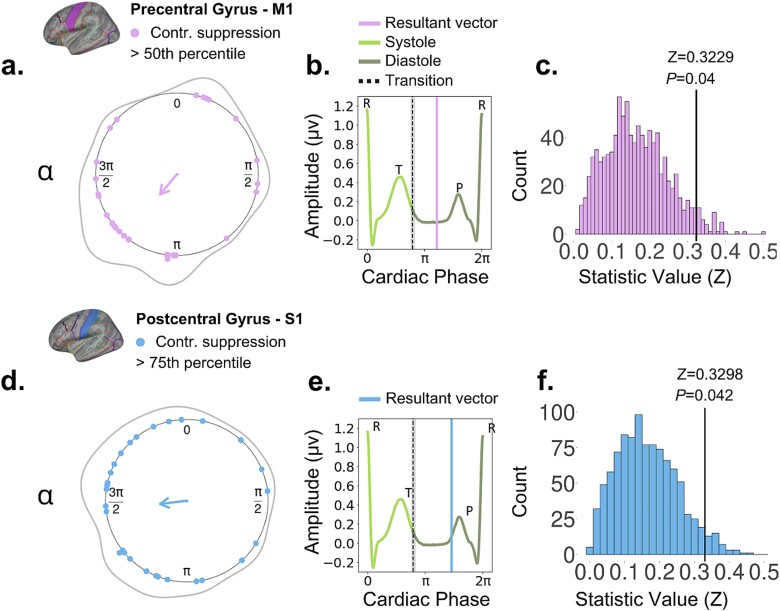
Temporal clustering along the cardiac cycle for cues associated with trials exhibiting pronounced contralateral suppression. We used circular statistics to determine if the latency of cues in trials with pronounced contralateral alpha and/or beta suppression clustered at a specific angle on the unit circle, representing the cardiac cycle. To identify these trials, we assessed three different power-thresholding values: 50%, 75%, and 90% of the trial-wise contralateral minus ipsilateral PSD distribution, separately for alpha and beta ranges, leading to a suppression index. The top and bottom panels represent results in the alpha range using the 50th and 75th percentiles, respectively. From left (a, d), the circular plots illustrate the mean resultant vector (group-level statistics) pointing to the angle at which clustering occurred. Individual dots indicate the subject-specific direction of the mean resultant vector. The Rayleigh test was significant in M1 with a 50th percentile threshold (a; *Z* = 0.3229, *P* = 0.04727), and in S1 with a 75th percentile threshold (d; *Z* = 0.3298, *P* = 0.0412), indicating a deviation from the uniform distribution. The middle panels (b, e) display the position of the group-level mean resultant vector relative to ventricular diastole (vertical purple line: top, blue line: bottom). The dashed line with shaded areas represents the group-average transition from systole to diastole (end of the T-wave). The right-side panels (c, f) present the empirical Rayleigh *Z* statistic alongside a null distribution of *Z* statistical values, estimated from 1000 Monte-Carlo-based permutations of randomly generated angles for each trial and participant. This approach involved subject-level analysis, followed by group-level analysis.

To identify trials with “pronounced suppression” we applied a criterion based on three different data embeddings: selecting trials that fell at the 50th, 75th, or 90th percentile of the suppression index distribution across all trials separately for each participant. First, at the individual participant level, the Rayleigh test did not reveal any significant effects across most participants. However, at the group level, a significant departure from a uniform distribution was observed in the M1 region for suppression index values exceeding the 50th percentile, associated with trials of “particularly pronounced” contralateral suppression in the alpha frequency range (Rayleigh’s Z = 0.3229, *P* = 0.04727; Fig. 7ab). The direction of the group-level mean resultant vector was 4.59 *radians*, within the diastole. In the S1 region, a significant deviation was only detected for contralateral alpha suppression above the 75th percentile (Rayleigh’s Z = 0.3298, *P* = 0.0412; Fig. 7de). This effect was associated with a mean resultant vector aligned at 3.84 *rad*, also placed within the diastole phase. Other combinations of percentile threshold embedding, ROI and frequency band did not yield significant results.

In a control analysis, we compared the empirical Rayleigh Z statistic corresponding to the group-level significant suppression index effects to a null distribution of Z statistical values based on 1000 Monte-Carlo-based permutations. This analysis revealed a significant effect in both cases (*P* = 0.04 for M1 and 50th percentile, *P* = 0.042 for S1 and 75th percentile). That means that the clustering of trials of particularly pronounced alpha or beta suppression at a specific angle during diastole was not explained by a random distribution of trial-related and subject-related angles on the unit circle.

## Discussion

Utilizing a MI task, where participants were instructed to mentally imagine the kinesthetic sensations of lifting their left and right thumbs, we found that the timing of the cardiac cycle modulates the contralateral alpha and beta suppression in sensorimotor cortices. This is the first study to demonstrate that MI performance—indexed by enhanced alpha and beta PSD suppression over the contralateral sensorimotor cortex—is significantly improved when the experimental cue instructing movement direction coincides with the diastolic phase, rather than the systolic phase. These results are in line with the baroreceptor hypothesis (Lacey 1967), also known as pulse inhibition hypothesis (Engelen et al. 2023), which proposes that heightened baroreceptor activation during systole leads to a generalized cortical inhibition and diminished response to sensory stimuli occurring in this cardiac phase. However, as discussed below, caution is advised regarding a mechanistic interpretation of these findings in relation to baroreceptor firing, as the evidence is indirect, and interpretations should consider methodological concerns (Caparco et al. 2024)*.* In the following, we discuss these findings and their potential application for the development of assistive and rehabilitative technologies, including methodological implementations that could improve MI-based BCIs and neurorehabilitation training, especially for poststroke patients.

### Contralateral alpha and beta suppression during MI

When analyzing neural responses to imagined movements, imagining kinesthetic sensations has been shown to result in attenuation of alpha and beta power in the hemisphere contralateral to the movement (Neuper et al. 2005; Sannelli et al. 2019). Using source reconstruction of the EEG epochs via LCMV beamforming and focusing on the primary motor and somatosensory cortices, we observed significantly more pronounced suppression of alpha and beta activity in the contralateral S1 and M1 regions during the MI task. In S1, the significant alpha suppression started at approximately 1500 ms postcue and lasted less than 100 ms, with no effects in M1. Similarly, suppression in the beta band was transient in both M1 and S1, appearing around 500 and 580 ms, respectively, and lasting less than 50 ms. Despite the transient nature of the significant effects, the findings align with the literature (Porro et al. 2000; Grèzes and Decety 2001; Hétu et al. 2013), indicating that imagining the sensation of movement activates cortical networks within S1 and M1.

By contrast, during actual ME, we did not find significant lateralization effects. Such absence of larger contralateral modulation during real motor performance is not uncommon. A recent review supports the notion that unilateral movements, such as wrist extension or thumb abduction as in our study, lead to bilateral cortical activation, with lateralization in such cases seeming to emerge with higher motor complexity (Chettouf et al. 2020). The authors concluded that this phenomenon could potentially be due to neural activation patterns involving both inhibition and excitation across the bilateral motor cortex during unilateral movements, with transcallosal tracts facilitating synchronous communication between hemispheres.

Supplementing the source-space analyses, sensor-level analyses also showed lateralization in the modulation of alpha and beta PSD during MI, although this was due to left-cued trials, with a more bilateral pattern observed in right-cued trials. No significant lateralization effects were observed in the sensor space during ME, consistent with the analysis in the source domain. The only partial presence of lateralization effects in alpha and beta suppression during MI in the sensor space may be due to high inter-subject variability in EEG responses during MI tasks. This variability can be attributed to differences in the strategies individuals use to imagine movements (Sannelli et al. 2019), as well as volume conduction effects in EEG, well described in early simulation work (Nunez et al. 1997), that reduce the apparent laterality of MI responses in sensor-level data (Tobón-Henao et al. 2022). This has prompted methodological developments within the BCI community, including spatial transformation techniques like common spatial patterns to optimally discriminate between types of imagined movements at an individual level (Blankertz et al. 2007). Alternatively, to mitigate factors contributing to variability in neural responses during MI, inverse modeling can be applied to reconstruct signals in the source domain (Tobón-Henao et al. 2022). We followed this later approach, implementing a validated pipeline using LCMV beamforming to source reconstruct neural activity in S1 and M1 for time–frequency domain analyses (Van Veen et al. 1997; Delorme et al. 2022).

### Cardiac cycle modulation

When testing our hypothesis that the cardiac cycle modulates neural responses during MI, analysis in the source space revealed that contralateral alpha and beta suppression in both M1 and S1 regions were more pronounced when the experimental cue occurred during the diastolic phase, with almost no effects observed for systole-cued trials. Specifically, the effects in diastole-cued trials were more sustained than those observed across the entire dataset, lasting from 0.5 to 2 s in both ROIs. Conversely, our results showed that during systole, there was a minimal lateralization effect in M1 that was short-lived, lasting only 60 ms.

Control EEG analyses matched the number of diastole and systole trials per subject, addressing potential trial imbalance concerns highlighted by previous research (Boudewyn et al. 2018). This confirmed the robustness of laterality effects in sensorimotor cortices, especially in S1, during diastole-cued trials, even with fewer diastole trials. However, in the full ‘joint’ dataset analysis, reducing the trial number led to a decrease in the temporal extent of the significant effects after adjusting for multiple comparisons. This indicates a particularly robust effect of diastole-timed cues on contralateral alpha and beta suppression during MI. Furthermore, using circular statistics, we found pronounced contralateral suppression clustered at specific times during the diastole phase: approximately at 4.59 radians after the T-wave offset in M1 and around 3.84 radians in S1.

These findings support the baroreceptor hypothesis (Lacey 1967), which posits that during systole, baroreceptors are maximally active and associated with a generalized inhibitory effect on cortical areas, reflecting heightened baroreceptor-mediated suppression (Duschek et al. 2013). In contrast, during diastole, baroreceptor activity is minimal, alleviating this inhibitory influence on the cortex. The opposite effects of baroreceptor stimulation on cortical activity during systole and diastole are further accompanied by changes in sensory processing, with enhanced sensory processing observed during diastole (Rau et al. 1993; Edwards et al. 2007; Duschek et al. 2013; Azzalini et al. 2019; Grund et al. 2022; Engelen et al. 2023).

Supporting evidence is provided by studies using experimental techniques such as neck-cuff procedures and phase-related external suction approaches to stimulate baroreceptor afferents (Rau et al. 1992; Droste et al. 1994). These methods have provided causal evidence that increased baroreceptor stimulation leads to decreased muscle tone, attenuated pain sensitivity, and reduced startle reflexes (Maixner 1991; Droste et al. 1994; Dworkin et al. 1994; Nyklíček et al. 2005; Duschek et al. 2013). Building on these findings, our study offers preliminary evidence that MI performance can be more effectively modulated by aligning cues or prompts with the diastolic phase of the cardiac cycle, when the inhibitory effects of baroreceptor activity are minimized.

Despite the widely acknowledged effect of baroreceptor stimulation on reducing cortical excitability and associated sensory processing attenuation (see Duschek et al. 2013 for a review), most studies contrasting the effects of systole and diastole on neural or behavioral responses lack direct evidence of this stimulation. Moreover, the effects of the cardiac cycle could be mediated by additional processes related to baroreceptor activity. For instance, during systole, the arterial baroreflex system modulates heart rate, vascular tone, and stroke volume to stabilize blood pressure fluctuations (Vaschillo et al. 2011). Concurrently, as blood is ejected during systole, the pulse wave travels through the arterial system, impacting mechanoreceptors in vascularized tissues, such as the glabrous skin (e.g. the palm of the hands and volar fingers; Macefield 2003). Similarly, arterial pulsations can drive muscle spindle discharge, with spindle firing often synchronized with the arterial pulse when background activity is absent (Birznieks et al. 2012). These processes align with the finding of decreased MSNA during systole, aiding in blood pressure stabilization (Wallin et al. 1975; Macefield 1998). Thus, while baroreceptor stimulation may have a causal role in some previously reported findings, and in our results, this effect could be indirect. Future research should aim to address this possibility.

Further contextualizing our results, current research highlights that pressure pulsatility—a key aspect of cardiovascular rhythms—strongly links cardio-respiratory and brain rhythmicity (Hamill 2023). This linkage suggests that rhythmic pressure fluctuations within the brain’s vascular system could synchronize neural networks across various regions, influencing brain functions and possibly modulating the brain’s responses to sensory and motor demands (Hamill 2023; Klimesch 2023). Accordingly, in the context of our study, differences in pressure pulsatility during systole and diastole could affect the synchronization or desynchronization of sensorimotor networks during MI, consequently modulating alpha and beta suppression in our task. Moreover, recent work has identified an additional, fast pathway for HBIs, demonstrating that pressure pulsations in olfactory bulb blood vessels, induced by the heartbeat, can directly influence the neuronal activity of a subset of mitral cells through the activation of mechanosensitive channels (Jammal Salameh et al. 2024). This is consistent with a fast baroreceptor transduction mechanism (Hamill 2023). Future research should aim to dissociate such mechanistic contributions from pressure pulsatility to the cardiac effects on perception, action, and cognition.

Regarding ME, our analyses did not reveal any significant effects of cardiac cycle phases on contralateral alpha and beta suppression. Recently, Al et al. (2023) observed that systole enhances neural responses during overt movements. In their study, both EMG activity and alpha and beta suppression were more pronounced during pinch movements when the onset coincided with systole, suggesting that systole enhances both peripheral and central neural activity. Conversely, our EEG analysis during overt ME did not identify any significant modulation by either systole or diastole on sensorimotor PSD suppression, thus precluding any inference about the role of the cardiac cycle in ME in our task. On the other hand, our analysis indicated increased peripheral EMG activity on the ipsilateral hand side when the ME cue was presented during diastole compared to systole, a finding replicated during MI. The discrepancies in EMG activity between our study and that by Al et al. could be due to the differences in how the analyses were time-locked: Al et al. locked their analysis to the pinch onset, while our study aligned EMG with cue presentation.

In our study, aligning EMG to the directional cue revealed relative ipsilateral muscle inhibition during systole and enhancement during diastole in both ME and MI conditions. The latencies of these effects in ME were around 1 and 1.8 s, contrasting with the findings from Al et al. (2023), where EMG effects were significant around 0.4 s postpinch movement onset. Physiological evidence suggests that muscle spindles, modulated by arterial pulsations, elicit one or two spikes at different cardiac cycle times in the absence of background activity (Birznieks et al. 2012). However, how this translates into sustained EMG activity during overt movements and whether EMG analyses locked to stimulus cues or motor responses would exhibit different patterns across the cardiac cycle remains unclear. Additionally, MSNA was shown to be inhibited by baroreceptor firing (Wallin et al. 1975; Macefield 1998). We suggest that future studies comparing the effects of systole and diastole on EMG activity during movement should model the effect of stimulus and response events on EMG simultaneously, accounting for variations in their latency on a trial-by-trial basis. Using general linear models, similar to those employed in neuroimaging and expanded to EEG analyses (Litvak et al. 2013), could advance this approach. This method would allow stimulus and response cues to be included as concurrent regressors influencing EMG activity.

In the EEG domain, we also recommend that future studies employ general linear models or related deconvolution approaches to simultaneously model the effects of various regressors, such as stimulus cues, response onsets, and parametric regressors like force output. This could help clarify the previously reported mixed findings in the motor domain. While further research is needed to better understand the dissociable effects of the cardiac cycle on action and perception, an emerging view suggests that systole impairs exteroceptive processing across multiple modalities but facilitates action. Conversely, diastole attenuates action while promoting active sensory sampling, thereby enhancing perception (Edwards et al. 2001; Edwards et al. 2002; Rae et al. 2018; Grund et al. 2022; Kunzendorf et al. 2019; Al et al. 2020; Galvez-Pol et al. 2020; Galvez-Pol et al. 2022; Skora et al. 2022; Al et al. 2023). However, precise analyses that dissociate the timing of the modulation from the timing of the event used for analysis will provide further insight into this hypothesis.

### Applications

In MI-based BCI training, users generate MI examples at specific times following system prompts, which is crucial for collecting labeled data to train supervised machine learning models (Sannelli et al. 2019). These models decode users’ intentions in real time during the BCIs’ communication phase (Sannelli et al. 2019). Our results suggest that monitoring the cardiac cycle in real time and delivering MI training cues during diastole, rather than systole, could enhance the detection of contralateral sensorimotor suppression. These findings have practical implications for assistive technologies, especially MI-based BCIs for individuals with severe neurological disorders and in neurorehabilitation for poststroke upper limb recovery, where detecting sensorimotor suppression remains a challenge (Ramos-Murguialday et al. 2013; Mansour et al. 2022).

Furthermore, recent findings suggest that HRV can enhance the decoding of neural signatures during oddball processing and MI in healthy participants, underscoring the potential of integrating cardiac information and HBIs to optimize MI-based BCI approaches (Kaufmann et al. 2012; Catrambone et al. 2021). This integration could lead to more precise and adaptive training protocols, providing more individualized BCI experiences.

Despite the difficulties in its detection, MI training significantly aids stroke survivors in regaining motor function. Daly and Bialocerkowski (2009) reported an 80% enhancement in motor control following MI training. Further studies highlight its benefits for reducing motor spasticity, increasing muscle strength, improving daily activities, and enhancing long-term muscle recovery (Ramos-Murguialday et al. 2013; Pichiorri et al. 2015; Biasiucci et al. 2018; Bai et al. 2020).

Our results advocate for dynamic neurorehabilitation protocols that leverage HBIs, suggesting that MI exercises cued during diastole could improve performance and kinesthetic sensations, potentially enhancing neural plasticity. This approach should initially be tested in healthy individuals with real-time cardiac monitoring and could later be applied to patients using offline analyses to track rehabilitation progress.

In relation to our observation that participants produced residual EMG activity during MI, it has been reported that many poststroke patients maintain some residual EMG activity following a cerebrovascular accident (Ramos-Murguialday et al. 2015). Nikulin et al. (2008) reported that alpha and beta suppression have similar spatial distributions in MI, with and without residual EMG, though the suppression is more pronounced when residual EMG is measurable. A future step could involve replicating our results in MI scenarios where muscular activity is undetectable by EMG, potentially employing an MI training protocol similar to that suggested by Nikulin et al. (2008).

### Limitations

Our study is not without its limitations. First, while we suggest that triggering MI trials during diastole may increase the engagement of the contralateral sensorimotor cortex and potentially improve MI performance through better kinesthetic sensations, we did not collect data on participants’ kinesthetic experiences. Future studies should replicate these findings using methods to quantify the vividness and kinesthetic sensations during MI, such as the Kinesthetic and Visual Imagery Questionnaire (KVIQ; Malouin et al. 2007; Yang et al. 2021). Incorporating such measures could elucidate the relationship between MI performance during different phases of the cardiac cycle and the vividness and sensation scores.

Second, for our results to be fully applicable in MI-based BCIs, they should also be replicated in an extended version of the current experimental paradigm that involves multiple sessions over several days and incorporates visual feedback to guide the learning process. In previous studies, multiple sessions and visual feedback demonstrated more concentrated desynchronized neural responses in the motor cortex (Rimbert and Fleck 2023) and promoted learning by enhancing a sense of agency and embodiment toward the task (Alimardani et al. 2018). Future studies could combine these two factors to deepen our understanding of how the cardiac cycle influences MI performance.

Furthermore, using a standard structural MRI template instead of subject-specific MRIs limits the precision of our source-reconstructed analyses. Previous research has shown that individual MRIs significantly enhance source localization accuracy (Huang et al. 2016). Employing individual MRIs in future studies could provide more detailed insights into the specific areas within M1 and S1 that are affected by diastolic modulation of alpha and beta suppression during MI.

## Conclusion

Our study highlights the significant influence of the cardiac cycle on MI performance, providing evidence that the diastole phase, characterized by minimal baroreceptor stimulation, may enhance the processing of experimental cues to guide imagined movements. While further studies are needed to ascertain whether this diastole advantage can be detected in real time and generalized to other imagined tasks, our findings contribute valuable insights into how the cardiac cycle affects MI performance variability. These insights hold promising implications for improving MI-based assistive technologies.

## Supplementary Material

Supplementary_Materials_final_bhae442

Supplementary_Materials_final_bhae442
