# Supplementary material for: Cardiac cycle modulates alpha and beta suppression during motor imagery
Source: Cereb Cortex. 2024 Nov 22;34(11):bhae442. doi: 10.1093/cercor/bhae442 (PMC11584698; doi:10.1093/cercor/bhae442)
Supplement: Supplementary_Materials_final_bhae442 [file supplementary_materials_final_bhae442.pdf]

Regress ECG

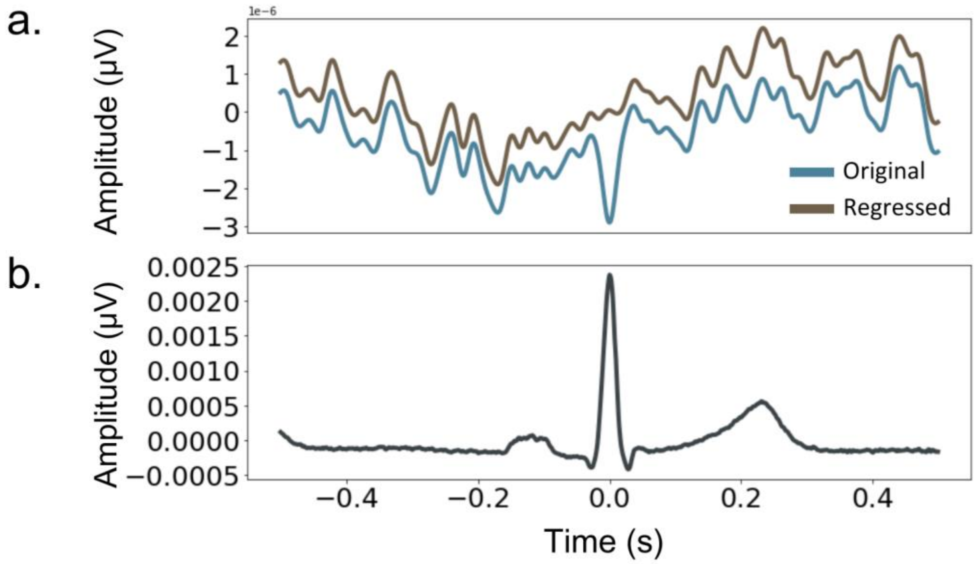

Figure S1. Removing the cardiac artefact using a regression-based method. a. Display of the R-peak locked event-related potential, ERP (in microvolts), before (original) and after (regressed) applying the regression-based method. The removal of the cardiac artefact is visible at time zero. While the original trace in (a) shows a negative deflection that coincides with the timing of the R-peak (shown in b), the regressed traces demonstrate the applied correction. For clarity, only the result of one participant in each task is shown. (b) ECG waveform in the same participant.

EMG Activity

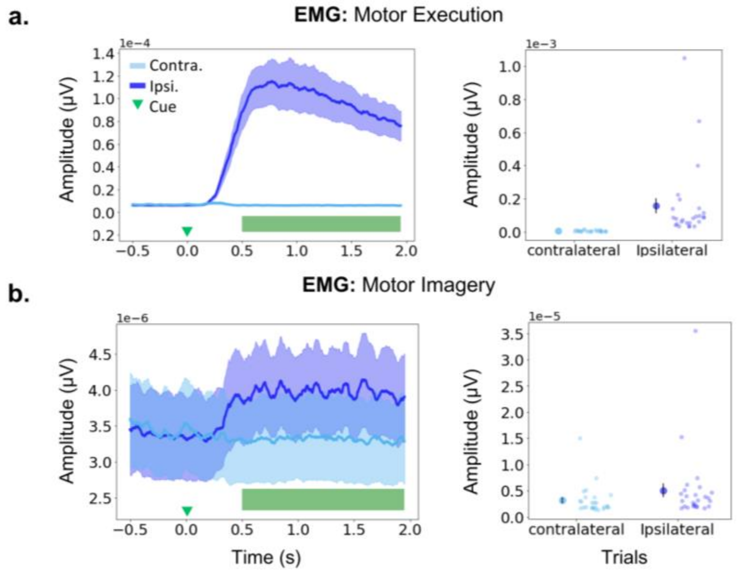

Figure S2. Differences between ipsilateral and contralateral EMG waveforms. Same as **Figure 3ab** in the main manuscript but including all n=26 participants for which EMG was available. Statistical differences are as reported in the main text.

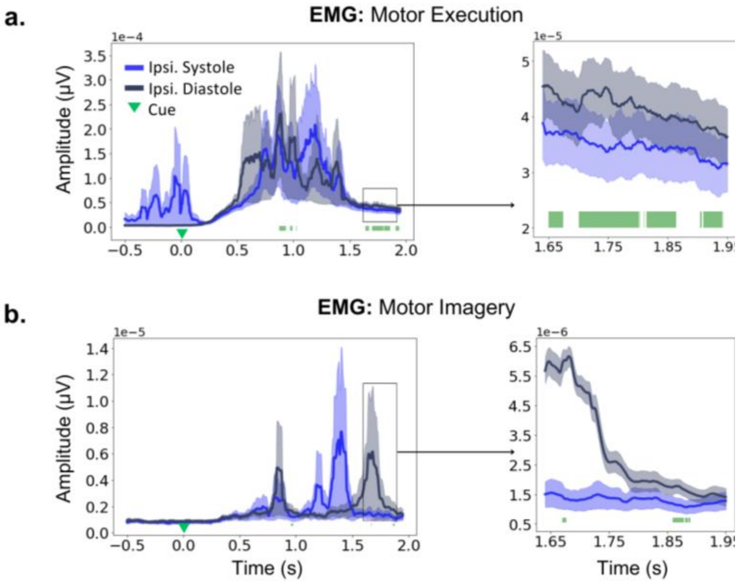

Figure S3. Differences between ipsilateral EMG waveforms for systole-cued and diastole-cued trials. Same as panels in Figure 3cd but including all n=26 participants for which EMG was available. Statistical differences are as reported in the main text and caption to Figure 3cd.

Source Analysis: Full-epoch Length

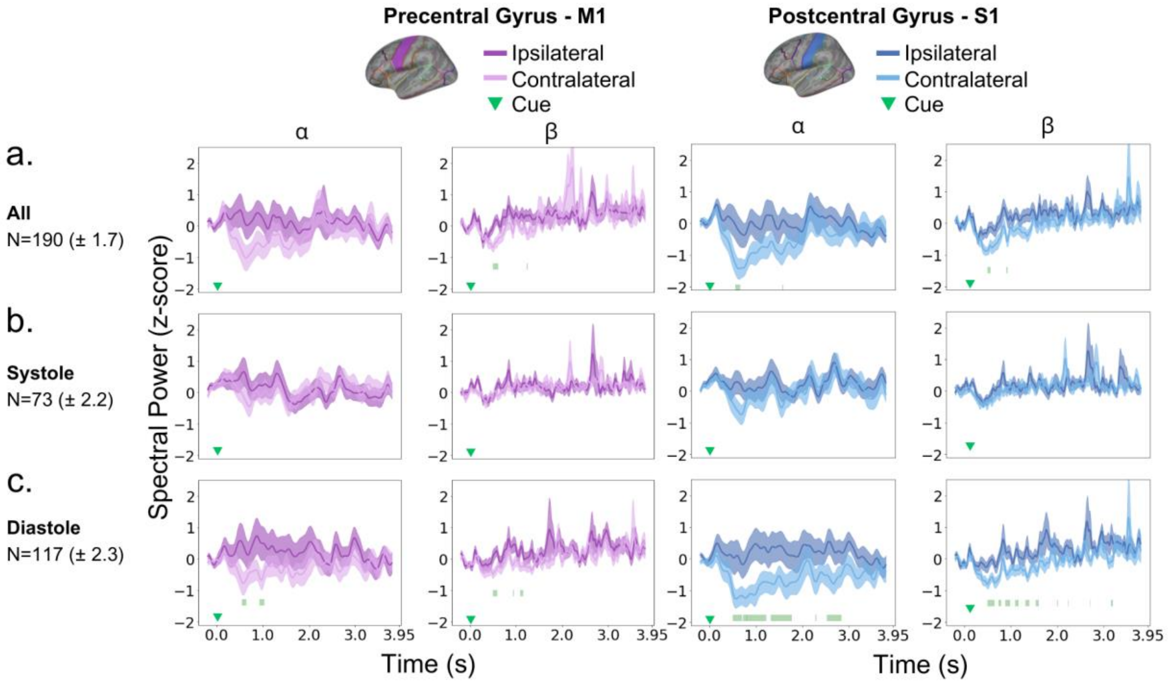

Figure S4. Differences between ipsilateral and contralateral power spectral density within the full-epoch length during motor imagery. Results of our exploratory analysis on source-reconstructed epochs from 0.5 to 4 seconds after the cue. The panels are similar to panels in Figure 4 but extending up to 4 seconds post-cue. Intervals associated with significant differences after FWER control are denoted by the green horizontal bars at the bottom.

Control Analysis on Source-reconstructed Epochs

Table S1. Control analysis in S1 for all trials. Contralateral versus ipsilateral suppression in alpha and beta bands in S1 and M1 was evaluated across subsets of trials (x10 times: 10 control runs) from the total set of trials, independent of cardiac phases, with the number of trials matched to the systole subset. The p-value ranges for statistical differences at each time point within the assessed window, 0.5–4 seconds, are provided. Significant effects after FDR control are marked with an asterisk *, and are accompanied by non-parametric effect sizes, Δ_{dep}.

Control runs	All Trials			
	**Alpha**		**Beta**	
	**P-value (P_{FDR})**	**Effect size (Δ_{dep})**	**P-value (P_{FDR})**	**Effect size (Δ_{dep})**
**1**	0.001–0.9926	/	0.0001–0.0001*	0.79
**2**	0.0006–0.0094*	0.76	0.0001–0.0072*	0.83
**3**	0.0268–0.0997	/	0.0012–0.9778	/
**4**	0.0001–0.0098*	0.86	0.0026–0.0102*	0.76
**5**	0.0002–0.0058*	0.86	0.0086–0.9742	/
**6**	0.0002–0.0008*	0.90	0.002–0.9988	/
**7**	0.0006–0.006*	0.76	0.0001–0.0038*	0.76
**8**	0.0532–0.951	/	0.001–0.991	/
**9**	0.0276–0.9862	/	0.0008–0.9966	/
**10**	0.0022–0.9946	/	0.0028–0.993	/

*Note: * Significant after FDR*

Table S2. Control analysis in S1 for all diastole-cued trials. Same as Table S1 but for subsets of diastole-cued trials, assessing the consistency of significant contralateral suppression when cues instructing movement direction occur in the diastolic phase of the cardiac cycle.

Control runs	Diastole			
	Alpha		Beta	
	**P-value (P_{FDR})**	**Effect size (Δ_{dep})**	**P-value (P_{FDR})**	**Effect size (Δ_{dep})**
**1**	0.0001–0.0372*	0.86	0.0008–0.0438*	0.86
**2**	0.0001–0.0254*	0.90	0.0001–0.0256*	0.83
**3**	0.0014–0.9986	/	0.001–0.9584	/
**4**	0.0001–0.0552*	0.83	0.0016–0.0524*	0.76
**5**	0.0001–0.0268*	0.76	0.0004–0.0292*	0.90
**6**	0.0004–0.0176*	0.80	0.0008–0.0196*	0.83
**7**	0.0001–0.0296*	0.83	0.0001–0.0336*	0.90
**8**	0.0001–0.0124*	0.79	0.0004–0.0132*	0.79
**9**	0.0001–0.0262*	0.90	0.0002–0.027*	0.83
**10**	0.0001–0.0342*	0.83	0.0008–0.0344*	0.83
*Note: * Significant after FDR*				

Table S3. Control analysis in M1 for all trials. Same as Table S1 but in M1.

Control runs	All Trials			
	Alpha		Beta	
	P-value (P_{FDR})	Effect size (Δ_{dep})	P-value (P_{FDR})	Effect size (Δ_{dep})
**1**	0.0022–0.9992	/	0.0004–0.9944	/
**2**	0.0032–0.0072*	0.76	0.0002–0.0092*	0.83
**3**	0.0014–1	/	0.0014–0.9998	/
**4**	0.0002–0.0104*	0.83	0.0004–0.0088*	0.72
**5**	0.0354–0.9508	/	0.0226–0.9972	/
**6**	0.0076–0.9742	/	0.0068–0.9778	/
**7**	0.0496–0.9946	/	0.0001–0.006*	0.79
**8**	0.037–0.9422	/	0.0001–0.9858	/
**9**	0.1192–0.9852	/	0.006–0.9998	/
**10**	0.0118–0.9914	/	0.0064–0.9948	/
*Note: * Significant after FDR*				

Table S4. Control analysis in M1 for diastole-cued trials. Same as Table S1 but for M1 and diastole-cued trials.

**Control runs**	**Diastole**			
	**Alpha**		**Beta**	
	**P-value (P_{FDR})**	**Effect size (Δ_{dep})**	**P-value (P_{FDR})**	**Effect size (Δ_{dep})**
**1**	0.0018–0.0424*	0.83	0.012–0.043*	0.79
**2**	0.0038–0.0256*	0.83	0.0158–0.0242*	0.83
**3**	0.0036–0.9966	/	0.0192–0.9924	/
**4**	0.0001–0.0476*	0.86	0.0084–0.0508*	0.72
**5**	0.004–0.0306*	0.76	0.0001–0.0304*	0.90
**6**	0.0006–0.02*	0.83	0.0114–0.0194*	0.59
**7**	0.0006–0.0336*	0.79	0.0016–0.032*	0.79
**8**	0.0012–0.0116*	0.72	0.0356–0.98	/
**9**	0.001–0.0266*	0.83	0.0158–0.0274*	0.69
**10**	0.0076–0.0346*	0.76	0.0018–0.0338*	0.79
*Note: * Significant after FDR*				

Sensor Analysis

Cluster-based permutation analysis was conducted to identify significant differences between the normalised PSD of left-cue and right-cue conditions across the scalp, aiming to find clusters associated with a lateralisation effect. If alpha and beta suppression demonstrates a lateralised effect, contrasting left-cued trials against right-cued trials (i.e., left-cued minus right-cued) should yield a combination of a negative cluster in the right hemisphere (contralateral suppression for left-cued trials) and a positive cluster in the left hemisphere (contralateral suppression of right-cued trials, reversed suppression due to the subtraction). This analysis was performed first for all ME and MI trials, followed by separate analyses for trials that occurred during the systolic and diastolic phases of the cardiac cycle within the main window of interest (0.5–2.0 seconds) and the full-epoch length (0.5–4.0 seconds).

For the ME task, no significant clusters were identified in the analysis of all trials (*P* adjusted for family wise error rate, P_{FWER}, in the range: 0.0299–0.940). Similarly, no significant clusters were found for trials cued during systole (P_{FWER} in range: 0.0470–0.9062) or during diastole (P_{FWER} in range: 0.0365–0.9122). In the MI task, while no significant clusters were found for trials cued during systole (P_{FWER} between 0.1517–0.9421), one significant positive cluster was identified when analysing trials across the entire cardiac cycle (P_{FWER}=0.002), and, similarly, for trials cued during diastole (P_{FWER}=0.014). In both cases, the positive cluster was in the left hemisphere (see **Figure S5**). While we expected this to indicate a larger contralateral suppression for right-cued than left-cued trials, separate topographic plots in each condition revealed that the positive cluster was explained by a more bilateral suppression in right-cued trials, and the expected contralateral suppression in left-cued trials. See below and **Figure S6**.

For all trials, and in the alpha frequency band, the effect was sustained and extended from 500 ms to 2000 ms post-stimulus, predominantly widespread over channels in the left hemisphere, but extending to a few frontocentral electrodes in the right hemisphere. In the beta frequency band, the significant effect was more transient and discontinuous than in the alpha band. The topographic plot indicates that the effect is prominently lateralised to the left-hemisphere (see **Figure S5a**). When cluster-based analysis was carried out with trials that occurred during the diastolic phase, the effect of the significant positive cluster was shorter than in all trials, extending from 500 to 1000 ms post-cue in both the alpha and the beta frequency bands. The topographies also demonstrated a widespread involvement of sensors in the left-hemisphere, with a few frontal sensors in the right-hemisphere. In the beta frequency band, the topographic spread of the effect was prominently left-lateralised (see **Figure S5b**).

The same cluster-based analysis was extended in the full-epoch length of 0.5–4.0 seconds post-cue. It confirmed the same results in ME and MI as in the 0.5–2.0 s analysis; no additional effects were observed after 2 seconds.

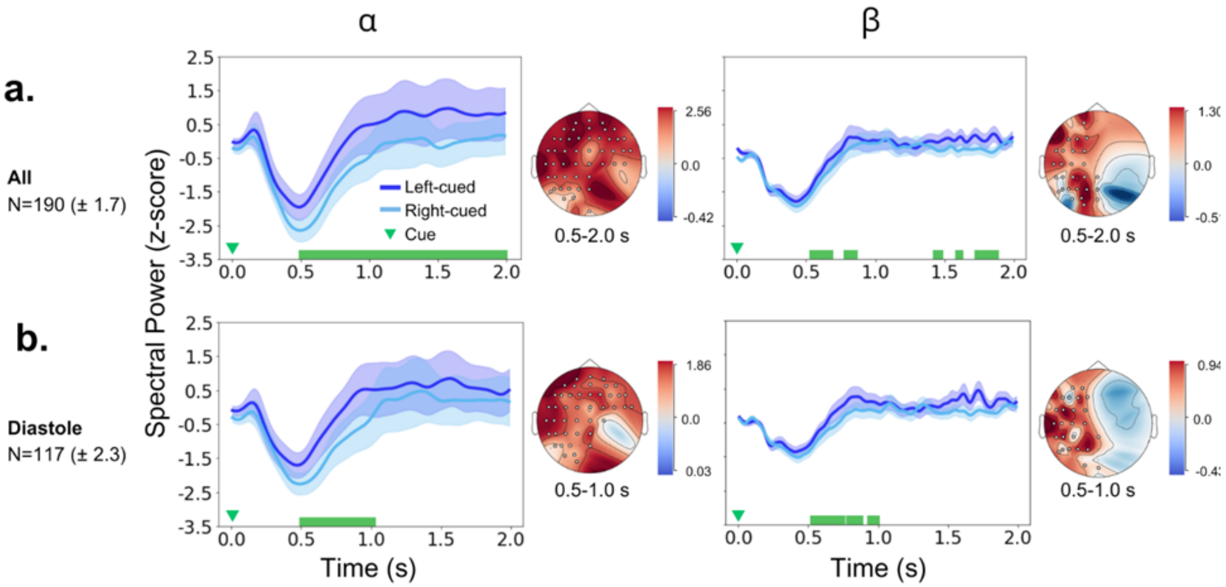

Figure S5. Results of the cluster-based permutation analysis for normalised PSD differences between left-cued and right-cued conditions during motor imagery. The figure shows the normalised PSD for the left-cued (dark blue) and right-cued (light blue) conditions plotted in the time window of interest (0.5–2.0 seconds). The darker lines indicate the group average, and the shaded areas represent ± 1 SEM. For each time-frequency plot, the corresponding topography is shown on the right-hand side, with the significant sensors marked in white. The colour bar indicates the minimum and maximum difference between the left-cued and right-cued traces, averaged in the significant time-window. (a) For all trials, a significant positive cluster was found in the alpha (left panel, α) and beta (right panel, β) frequency bands (P_{FWER}=0.002). The topographic plots show sustained effects from 500 to 2000 ms post-stimulus in the alpha band and more transient effects in the beta band, predominantly over the left hemisphere. (b) For trials that occurred during diastole, a significant positive cluster (P_{FWER}=0.014) involving both frequency bands was observed from 500 to 1000 ms post-cue, with a similar topographic distribution as all trials, but with a more restricted temporal window.

As mentioned above, plotting the topography of the alpha and beta suppression separately for right-cued and left-cued trials suggested that the distribution was lateralised to the contralateral side, as

expected, for left-cued trials, while the pattern was more bilateral for right-cued trials, likely reflecting volume conduction effects (**Figure S6**).

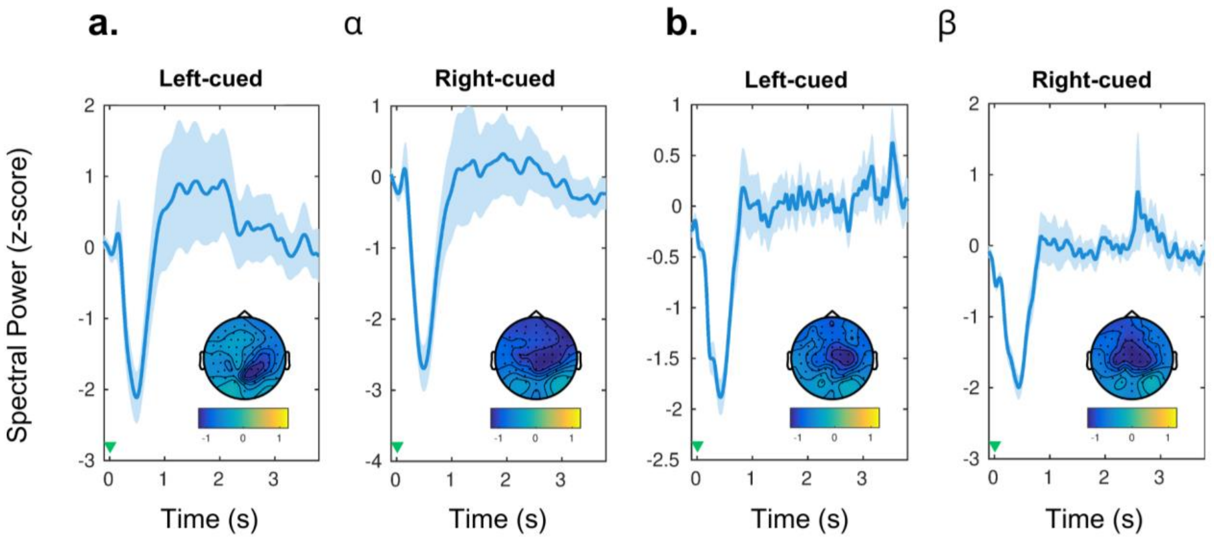

Figure S6. Time course and topography of normalised alpha and beta PSD changes following left- or right-side cues. The time course of the suppression in each frequency band and cue condition is displayed as mean (blue bold line) and SEM (shaded area). The time course represents the mean across all channels, followed by the average across participants (with associated SEM). Insets display the topographic distribution, averaging the normalised PSD within the range of the most pronounced suppression between 0.2–1 seconds. The reduction in normalised PSD was lateralised to the contralateral hemisphere, as expected, for left-cued trials, but the pattern was more bilateral for right-cued trials.

Because **Figure S6** revealed that the most pronounced suppression occurred from 0.2–1 seconds post-cue during MI, we conducted an exploratory analysis in this specific window. We assessed whether alpha and beta suppression in selected sensorimotor channels from the contralateral hemisphere was more pronounced than in the ipsilateral hemisphere for directional cues presented during systole or, separately, diastole. We selected channels commonly used for sensorimotor alpha and beta oscillations in EEG research: FC1, FC3, FC5, C1, C3, C5, CP1, CP3, CP5 in the left hemisphere, marked as contralateral for right-cued trials; and the analogous channels in the

right hemisphere, FC2, ..., CP6, as contralateral for left-cued trials. Statistical analysis contrasting contralateral and ipsilateral alpha and beta suppression between trials cued in the systolic phase revealed no significant differences (P > 0.05, permutation test with 5000 permutations; **Figure S7a**). By contrast, when trials were presented in the diastolic phase, the contralateral beta suppression was more pronounced as compared to the ipsilateral suppression (P < 0.01, uncorrected exploratory analysis; between 0.6–0.7 seconds). See **Figure S7b**. No effects in alpha were observed.

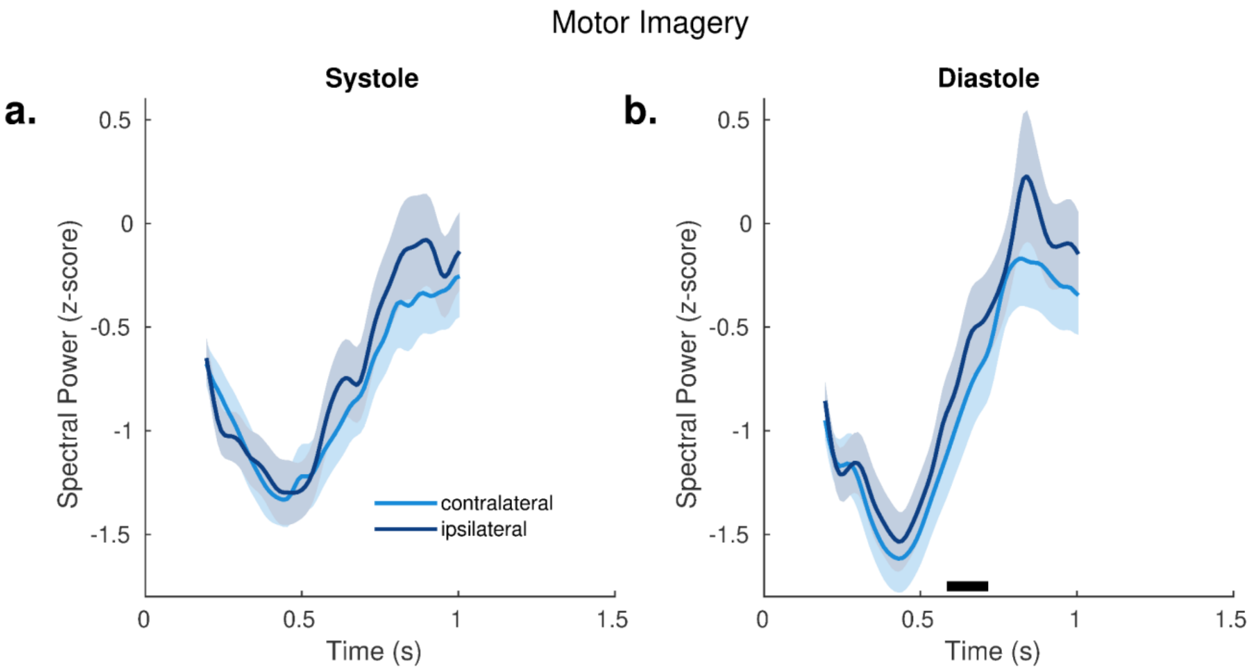

Figure S7. Time course of normalised beta PSD in selected contralateral and ipsilateral EEG channels (see main text) for cues timed with the systolic (a) or diastolic phase (b). The bold line denotes mean, while shaded areas denote SEM. Black line at the bottom denotes statistical differences observed during diastole (P < 0.01, uncorrected).

Lastly, we repeated the same exploratory analyses as shown in **Figures S6** and **S7** for the ME task, to understand potential factors contributing to the lack of significant differences in alpha and beta suppression between left-cued and right-cued trials. As depicted in **Figure S8**, and aligning with

the MI pattern, the suppression in the alpha and beta bands was more pronounced from 0.2 to 1 second and was prominently lateralised to the contralateral hemisphere for left-cued trials, but not for right-cued trials (**Figure S8**). Furthermore, directly assessing the differences between contralateral and ipsilateral channels in the normalised alpha and beta PSD, separately for epochs cued during systole or diastole revealed no significant differences ($P > 0.05$; **Figure S9**). This contrasts with the cardiac cycle effects on sensor-space contralateral suppression observed during MI (**Figure S7**).

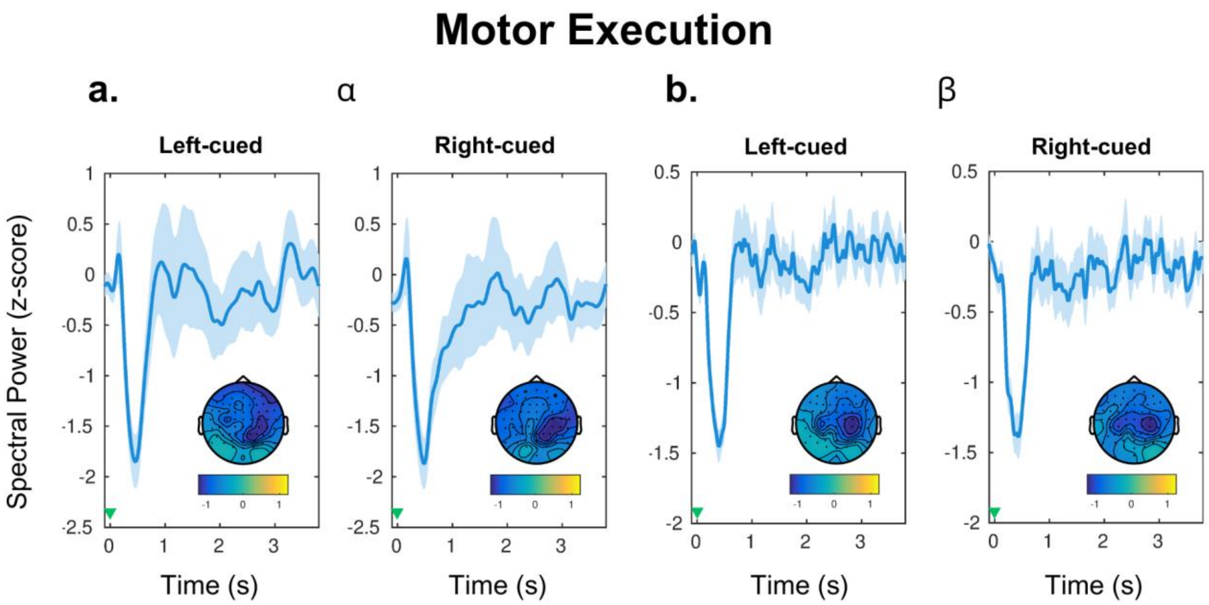

Figure S8. Same as **Figure S6** but during motor execution.

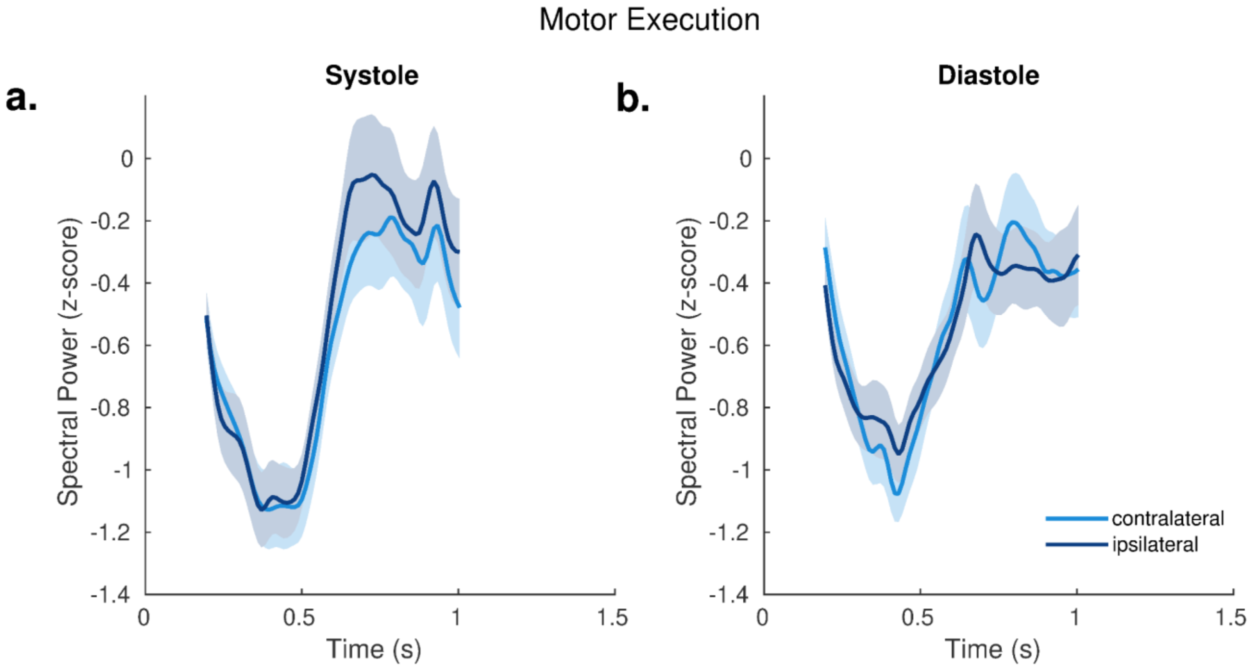

Figure S9. Same as **Figure S7** but during motor execution.
